# Gender gaps at the academies

**DOI:** 10.1073/pnas.2212421120

**Published:** 2023-01-19

**Authors:** David Card, Stefano DellaVigna, Patricia Funk, Nagore Iriberri

**Affiliations:** ^a^Department of Economics, University of California, Berkeley, CA 94720; ^b^National Bureau of Economic Research, Cambridge, MA 02138; ^c^Department of Economics, Università della Svizzera italiana, 6900 Lugano, Switzerland; ^d^Department of Economic Analysis, University of the Basque Country UPV/EHU, 48015 Bilbao Spain; ^e^IKERBASQUE, Basque Foundation for Science, 48009 Bilbao, Spain

**Keywords:** honorary society, gender gaps, Matilda effect

## Abstract

Historically, a large majority of newly elected members of the National Academy of Science (NAS) and the American Academy of Arts and Science (AAAS) were men. Within the past two decades, however, that situation has changed, and in the last 3 y, women made up about 40% of the new members in both academies. We build lists of active scholars from publications in the top journals in three fields—psychology, mathematics, and economics—and develop a series of models to compare changes in the probability of selection of women as members of the NAS and AAAS from the 1960s to today, controlling for publications and citations. In the early years of our sample, women were less likely to be selected as members than men with similar records. By the 1990s, the selection process at both academies was approximately gender neutral, conditional on publications and citations. In the past 20 y, however, a positive preference for female members has emerged and strengthened in all three fields. Currently, women are 3 to 15 times more likely to be selected as members of the AAAS and NAS than men with similar publication and citation records. The positive preference for women may be in part a reflection of concerns that women face higher barriers to publishing in top journals and may receive less credit for their work.

## Significance

The 2007 Beyond Bias and Barriers report from the National Academies of Science, Engineering, and Medicine asked honorary societies to “review their nomination and election processes to address the underrepresentation of women in their memberships.” We use data on publications and citations for scholars in psychology, mathematics, and economics to define the pipelines of scholars in the three fields and measure gender disparities in membership at the National Academy of Science and the American Academy of Arts and Sciences. There have been substantial increases since 1960 in the share of women in the pipelines of all three fields and even greater increases in the share of women newly elected to the two academies in all three fields.

Do female researchers achieve the same recognition and rewards as their male colleagues? This is a critical and timely question, given instances of discrimination both inside and outside academia (1–5) and the ideal of moving toward a gender-equal world. Underrepresentation of women has long been a concern in science (6), particularly in the fields with a strong mathematical orientation (7, 8) but also in non-STEM fields (9).

Assessing gender differences in scholarly recognition requires detailed information on the qualifications and achievements of different researchers. At the early stages of a research career, such data are largely unavailable. By the later stages of a career, however, most scholars have amassed an observable record that summarizes their primary research contributions. In this paper, we therefore focus on recognition received relatively late in the academic career, where women have also been underrepresented historically, see, e.g., refs. (10–13). Specifically, we study gender gaps in the election of new members to two of the oldest and most prestigious academies in the United States, the American Academy of Arts and Sciences (AAAS) and the National Academy of Sciences (NAS).

Each year, current academy members nominate and then elect a small number of individuals to become new members. The list of nominees is confidential, so in this study, we focus on overall selection probabilities for women and men who are not yet members. Since new members are typically 20 or more years into their careers, there is an ample record of publications and citations to compare candidates and evaluate gender differences conditional on the observed record. Moreover, given the long history of these academies, we are able to study trends in the gender gaps in selection from the early 1960s to today.

We propose and implement a 5-step procedure to measure gender gaps in the member selection process in three fields: psychology, economics, and mathematics. We begin by using the CVs of elected members of the two academies to identify the 13 to 16 highest impact journals in each field. We then construct a list of all articles published in these “top journals” and their associated citations in each year. Next, we extract the authors of these publications and build annual CVs for active researchers in each field that contain their cumulative publications and citations as of that year. We then use a combination of first-name matching and hand searches to assign gender to active researchers. And finally, we combine our annual CVs with information on the newly elected members of each academy in each year from 1960 on. The resulting data set allows us to identify the probability of selection as a member of a given academy from among the “risk set” of active researchers who are not yet members in each year.

This procedure, which builds on our previous work on honors in economics in ref. (14), has a number of advantages. First, it provides a simple method of identifying potential candidates for membership of the two academies. For the three fields we study, over 92% of new members are in our journal-based risk set. Second, it yields plausible measures of research productivity, based on publications and citations, which we show are highly predictive of the probability of selection as a new member in the three fields of interest. Third, it allows us to build models of the selection process that vary over time, reflecting differences in the weights assigned to different journals (and potentially different subfields). The main disadvantage is that it relies on journal publications and is less well suited to book-based fields or those that use conference proceedings to disseminate research. We therefore focus on three fields that are broadly representative of those in the AAAS and NAS, that mainly rely on journal publications as outlets for scholarly work, and have widely different shares of female scholars: psychology, economics, and mathematics.

**Fig. 1.**
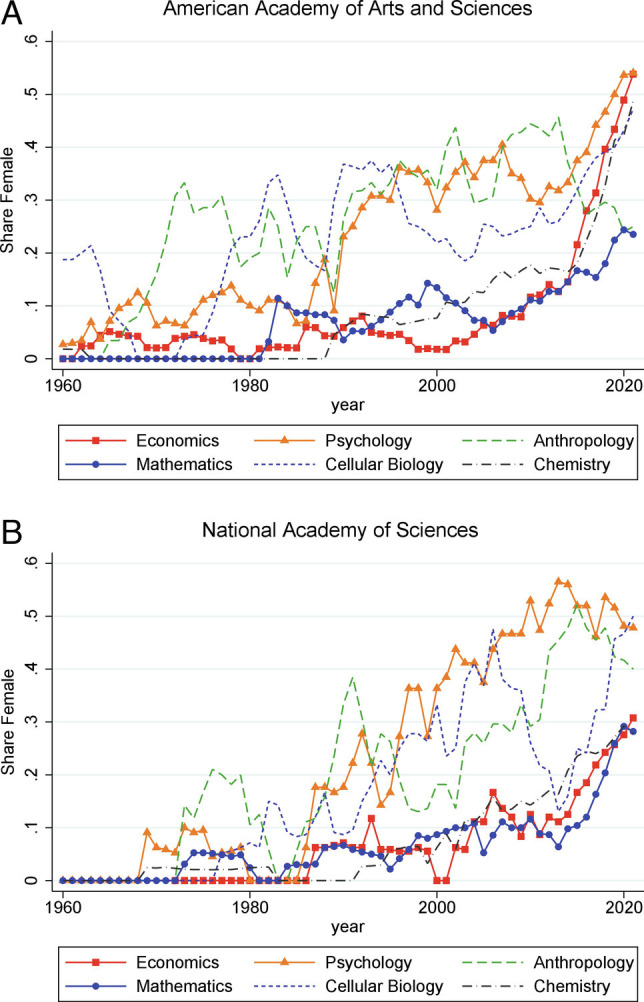
Share female of new members in different fields of AAAS and NAS. *Notes.* In this figure, we plot the share female among new members in AAAS and NAS. The shares are a moving average that includes two leads, the index year, and four lags. In anthropology, chemistry, and cellular biology, we gender algorithmically; in economics, mathematics, and psychology, we use a combination of algorithmic gender assignment and hand checking.

To put this choice of fields in context, Fig. 1 plots the evolution of the female share among newly elected members in six of the largest fields covered by the AAAS and NAS. For each of the fields, we plot a 7-y moving average of the female share of newly elected members. Prior to the 1980s, the female share was below 10% in most fields (especially at the NAS), though in anthropology, it was closer to 20%. Since the 1990s, the female share in psychology has been at or near the top among the six fields, with women representing close to half of new members inducted into the NAS in psychology since 2010. At the opposite end of the spectrum, economics and mathematics had historically low female representation, with patterns that are quite similar to those for chemistry. In the last 5 to 10 y, however, both disciplines have seen a sharp increase in representation. The rise is especially striking for economics at the AAAS, where over 50% of new members in 2021 were female. Cellular biology, by comparison, typically lies between the two groups of disciplines.

The three fields of focus therefore include one field that is on the higher end of female representation—psychology—one at the bottom end—mathematics—and one that was historically at the bottom but has recently caught up, at least in AAAS—economics. What accounts for these patterns and these changes? These aggregate statistics, as interesting as they are, cannot tell us whether the differences reflect changes in the pipeline of candidates or differences in the probability of selection of female candidates, controlling for measures of research productivity. Our analysis allows us to disentangle these differences and quantify the magnitude of any gender gaps in the probability of election, conditional on academic achievement. We present our key results in *Results*, after briefly discussing the data and summary statistics in *Data and Sample Construction*.

This study has some typical features of the scholarly work of Dr. David Card, who has chosen this as his inaugural article. The aim is to produce systematic empirical evidence on a topic of societal importance—gender differences in treatment—even in cases in which experimental evidence is not available. In this case, the construction of a systematic sample of candidates for NAS and AAAS election enables the comparison between the academic record of elected candidates and nonelected ones, and thus inference on gender differences. This article builds on previous work of David Card with collaborators Stefano DellaVigna, Patricia Funk, and Nagore Iriberri on gender differences in the review process in economics (15) and on the election of economists to fellowships such as the Econometric Society (14).

Before proceeding, we note three important caveats to our findings. First, comparisons of election probabilities between male and female researchers with similar publications and citations implicitly assume that male and female researchers face the same standards for publication and are equally likely to be cited conditional on the quality of their work. If female researchers face a higher bar for publishing their work, or are less likely to be cited (5, 15–17), but voters who are selecting new members at the academies take these differences into consideration, we will tend to find a gender gap in favor of female researchers. Second, election to the AAAS and the NAS in particular is mainly limited to United States-based researchers, and is driven by the preferences of United States-based members. Patterns in other regions of the world may differ. Third, the gender differences we document at the later stages of scholars’ careers may not reflect differences at earlier stages, such as at the initial appointment or promotion stage. Indeed, if women face barriers at the early career stage, e.g., refs. (18) and (19), then the set of active scholars we study may be differentially selected by gender and may overrepresent especially talented female researchers.

## Data and Sample Construction

### Sample.

We outline here the five steps of the sample construction, with additional details in *SI Appendix*.

In step 1, we identify the highest-impact journals in each field. Since conventional impact factors are hard to compare and not always reliable even within a field, we instead identify the most cited publications of members of the two academies in a given field, and select the journals that most often appear in this list. Specifically, for all AAAS and NAS members with a Google Scholar profile page, we identify their 20 most-cited publications and tabulate the journals with the most articles in this list. This provides us with a revealed-choice ranking of journals in a given field; we select the top 13 to 16 journals in this list (depending on the field) as the “top journals” for that field (*SI Appendix*).

Reassuringly, the selected journals include most that would be selected by other ranking criteria. For example, in psychology, the top two journals in this list are the *Journal of Personality and Social Psychology* and *Psychological Review*, two of the highest-impact journals in the field. Some of the other journals cover important field areas, such as *Child Development*, or represent broad outlets, such as *Psychological Science*. In economics, the first 5 journals on the list are the so-called “top 5” (see ref. (20)): the *American Economic Review*, *Econometrica*, the *Quarterly Journal of Economics*, the *Journal of Political Economy*, and the *Review of Economic Studies* (in that order). The others include top field journals such as the *Journal of Economic Theory*. In mathematics, the first three journals on the list are the *Annals of Mathematics*, *Communications on Pure and Applied Mathematics*, and *Communication in Mathematical Physics*, followed by a mixture of pure math journals, statistics journals, and mathematical physics. We note that the set of influential journals within each field would look very similar if we used only the publications of members of either academy, suggesting a common ranking process.

In step 2, we download the titles and author names for all the articles published in the top journals of each field up to the end of 2019. We eliminate notes, letters to the editor, and articles of less than three pages. For each remaining article, we merge on the number of citations to that article (from Web of Science) in each year subsequent to publication. We retain all articles published since 1930 to allow for at least a 30-y record of publications for the earliest members studied in our sample (who were elected as early as 1960).

In step 3, we build synthetic annual CVs for researchers who were listed as an author of one or more of the selected articles. Specifically, we link all researchers with the same first, middle, and last names to create a unique author record. For each author (i.e., unique name), we then create a CV for each year since the author’s first publication in one of the top journals. This CV includes the cumulative number of publications by the author in each journal up to year *t* and the cumulative citations to previously published articles in each of the top journals up to year *t*. For example, an entry would be David E. Card, year 2007: 5 articles in the *American Economic Review*, 5 articles in *Econometrica*, 301 citations to articles published in the *American Economic Review*, 410 citations to articles published in *Econometrica*, etc. We include individuals in the sample for 18 y since their last publication, and we remove individuals if we locate a year of death in Wikipedia. We describe in *SI Appendix* a number of checks that we implement to ensure that we are combining publication records for people who use different forms of their name (e.g., with and without a middle initial). For simplicity, we refer to the set of selected authors with at least one prior publication in one of the top journals in their field as the set of active publishers as of a given year.

In step 4, we assign a gender to the authors identified in the previous step. We begin by using standard name lists for the United States and Germany to assign gender whenever a name is nearly always one gender or another, as in ref. (15). This first pass still leaves many authors ungendered. A team of research assistants then assigned gender to as many remaining names as possible, prioritizing researchers with more publications. We outline the procedure in detail in *SI Appendix*.

In step 5, we match the data set of annual CVs to the list of newly selected members of the AAAS and NAS in each year for the three fields of interest. For the AAAS, we identify members as those listed on the AAAS website with specialties in “Economics,” “Psychology,” and “Mathematics, Applied Mathematics, and Statistics.” The website is missing some members selected in the earlier years of our sample, so we add information from the AAAS Book of Members, as explained in detail in *SI Appendix*. For the NAS, we include members listed on the NAS website with their primary field in the three fields of interest.

### Summary Statistics.

Before we turn to the results, we present some summary statistics in Table 1, with additional details in the *SI Appendix*. For each field, our 5-step procedure yields an author-year sample of people who have previously published at least one paper in one of the top journals in that field. Characteristics of this sample, by field and 20-y subperiod, are presented in *Panel A* of the Table. In the first two decades of our sample (1960 to 1979), we have 148,608 author-year observations in psychology, with 14,771 different authors. In the last two decades (2000 to 2019), the psychology sample has 435,187 author-year observations with 43,982 different authors. Economics has less than half as many authors as psychology (but grew more quickly over our sample period), while mathematics is more similar in size (and also grew more quickly than psychology).

**Table 1.** Summary statistics

	Psychology			Economics			Mathematics		
	1960 to 1979	1980 to 1999	2000 to 2019	1960 to 1979	1980 to 1999	2000 to 2019	1960 to 1979	1980 to 1999	2000 to 2019
*Panel A. All publishers*									
No. of author-years	148,608	305,345	435,187	60,163	148,007	230,080	129,817	311,824	555,438
Unique authors	14,771	26,614	43,982	6,314	12,388	20,292	13,104	27,577	50,743
No. of pubs. in sample, Average	2.27	2.47	2.8	2.67	3.21	3.82	3.01	2.8	2.91
Cumulative citations, Average	24.76	59.53	198.63	10.9	49.84	202.45	14.85	34.2	80.53
Percent female	19.18	31.63	42.98	4.03	7.08	14.69	3.63	4.45	7.19
Percent unknown gender	10.6	9.3	8.7	8.8	5.41	2.07	26.48	29.23	28.69
Years since first publication, Average	8.24	11.53	11.47	10.18	11.24	13.04	9.21	11.35	12.15
*Panel B. AAAS members*									
New AAAS members (matched)	56	70	106	107	136	167	103	130	144
No. of pubs. in sample, Average	6.93	13.6	26.39	9.08	16.06	18.77	15.13	13.37	17.65
Cumulative citations, Average	195.11	877.61	2996.94	60.5	530.63	1458.14	161.82	307.65	797.1
Percent female	7.14	25.71	39.62	1.87	3.68	15.57	0	9.23	11.11
Percent unknown gender	0	0	0	0	0	0	0	0	0
Unmatched member, Male	7	4	5	16	3	0	13	6	2
Unmatched member, Female	1	2	0	1	0	0	0	1	1
Years since first publication, Average	17.8	24.4	31.36	20.78	20.9	22.38	17.75	23.1	26.46
*Panel C. NAS members*										
New NAS members (matched)	40	32	55	27	38	48	75	97	115
No. of pubs. in sample, Average	10.63	11.72	23.53	16.33	17.5	29.1	17.19	15.05	19.15
Cumulative citations, Average	364.43	925.78	3362.04	246.85	778.32	3770.73	205.13	368.86	1307.42
Percent female	5	15.63	49.09	0	5.26	14.58	2.67	5.15	12.17
Percent unknown gender	0	0	0	0	0	0	0	0	0
Unmatched member, Male	1	4	2	1	0	0	6	5	3
Unmatched member, Female	0	2	0	0	0	0	0	0	2
Years since first publication, Average	18.95	23.53	30.2	26	29.87	32.5	21.8	24.52	27.89

Notes: In this table, we present summary statistics across psychology, economics, and mathematics. We drop observations where the years since last publication is greater than 18.

The average researcher in the sample has a relatively small number of publications, e.g., 2.27 in psychology in 1960 to 1979, and a modest number of citations, e.g., 25 cumulative citations for psychologists in the earliest decades. Numbers of citations have risen in all three fields, reflecting trends toward more citations per published paper, more journals in each field, and broader coverage of Web of Science. Consistent with Fig. 1, the share of females in the sample of active publishers is much larger in psychology than in either mathematics or economics and also grew relatively more in economics than mathematics. Despite our best efforts, we are unable to assign gender for 9 to 10% of psychologists, 2 to 9% of economists, and 26 to 29% of mathematicians. The higher share with unknown gender in mathematics reflects the tendency in that field to use initials rather than full names and the much higher representation of Asian researchers, whose first names often cannot be assigned a unique gender. Individuals of unknown gender are not included in the analysis below. Finally, the average number of years since first publication ranges from 8 to 13 y and is rising across the sample period in all three fields.

Panels *B* and *C* of the table show the characteristics of newly selected members of the AAAS (*Panel B*) and NAS members (*Panel C*) in each discipline and subperiod. We note that the number of newly elected members is fairly small: about 3 to 5 per year in the AAAS for psychology, versus 5 to 8 per year in the AAAS for economics and mathematics, and 1 to 3 per year in the NAS for both economics and psychology, versus 4 to 6 per year in mathematics. These elected members have, not surprisingly, much higher numbers of publications and citations than the overall population of active publishers: 3 to 10 times more publications and 6 to 15 times more citations. For example, the average number of publications in the high-impact journals in our sample for AAAS members in 2000 to 2019 is 26.39 in psychology, 18.77 in economics, and 17.65 in mathematics.

The share of women among academy members was very low in the earliest decades of our sample—in fact zero for economics in the NAS and mathematics in the AAAS—but has grown substantially in recent decades, as noted in Fig. 1. We are able to assign gender to 100% of newly selected members of both academies. Across the three fields, the number of members that does not match to the sample of active publishers is small, especially in the last two decades, e.g., for the 2000 to 2019 decades summing across AAAS and NAS, just 7 researchers in psychology, 0 in economics, and 8 in mathematics. These members, who are not included in the analysis below, have atypical research outputs, such as book publications (most often in psychology) or publications in neighboring disciplines or occasionally public service distinctions. We note that including these members would not substantially alter the conclusions about the gender composition, since Table 1 shows that their gender composition tracks that of members who are matched to the sample. Finally, across all three fields, there is a tendency for the number of years between first publication and selection as an Academy member to rise during our sample period: from around 18 to 20 y in the 1960 to 1979 period (with the notable exception of economics at NAS) to around 30 y in the 2000 to 2019 period (with the notable exception of economics at the AAAS, where the average was only 22).

## Results

Fig. 1 shows that there are large and persistent differences across fields in the female share of newly elected members of the AAAS and NAS. There are also notable changes over time within fields, sometimes occurring quite rapidly. Differences in the female share of new members can arise from two sources: differences in the female share of potentially qualified candidates for membership (i.e., the fraction of women “in the pipeline”) or differences in the probability of selection between equally qualified men and women.

To provide some preliminary evidence on the first explanation, we used our samples of active publishers to construct three potential pipelines of candidates for membership in the two academies. The first includes anyone with at least one publication in the set of top journals who is not yet a member of the particular academy. As we documented in Table 2; however, a typical newly selected member of either academy has 15 to 25 top journal publications (depending on the field and the time period) in the year of their selection. We thus form two additional pipeline samples: researchers with at least five publications in year *t* and researchers with at least 15 publications in year *t* who are not yet members of the respective academy. The latter group is arguably most comparable to newly elected members of the academies.

**Table 2.** Gender coefficients across fields and time

	Psychology		Economics		Mathematics	
Percent female (active publishers in 2010 to 2019)	48.22%		16.72%		10.85%	
Logit regression for selection as Member in Year t	AAAS	NAS	AAAS	NAS	AAAS	NAS
*Panel A (Parsimonious)*						
Female coefficient	-0.278	-0.722	0.116	–	–	0.624
1960 to 1979	(0.515)	(0.728)	(0.619)	–	–	(0.726)
Female coefficient	-0.120	-0.528	1.569^***^	1.846^*^	0.961	0.175
1980 to 1989	(0.617)	(0.732)	(0.593)	(1.034)	(0.594)	(1.018)
Female coefficient	0.561^*^	0.183	0.556	1.508	1.493^***^	1.166^**^
1990 to 1999	(0.317)	(0.666)	(0.712)	(1.008)	(0.351)	(0.513)
Female coefficient	0.529^*^	0.730^*^	0.917^**^	1.879^**^	0.360	1.170^**^
2000 to 2009	(0.292)	(0.440)	(0.434)	(0.763)	(0.511)	(0.470)
Female coefficient	0.419	1.056^***^	1.928^***^	1.897^***^	1.224^***^	1.173^***^
2010 to 2019	(0.290)	(0.364)	(0.276)	(0.518)	(0.320)	(0.371)
Cumulative citations	0.849^***^	0.932^***^	1.195^***^	1.458^***^	0.984^***^	1.160^***^
(inverse hyp. sine)	(0.093)	(0.124)	(0.064)	(0.114)	(0.053)	(0.061)
Cumulative publications	0.045^***^	0.036^***^	0.066^***^	0.031^***^	0.013^***^	0.013^**^
(number)	(0.009)	(0.009)	(0.008)	(0.008)	(0.005)	(0.005)
N	790,905	749,708	410,778	397,990	699,035	706,060
Pseudo R-squared	0.215	0.204	0.303	0.337	0.196	0.226
*Panel B (Full specification)*						
Female coefficient	-0.738	-0.701	-0.073	–	–	0.771
1960 to 1979	(0.512)	(0.756)	(0.635)	–	–	(0.752)
Female coefficient	-0.069	-0.496	1.803^***^	2.097^*^	1.246^**^	0.201
1980 to 1989	(0.630)	(0.758)	(0.634)	(1.094)	(0.592)	(1.112)
Female coefficient	0.702^*^	0.480	0.625	1.805^*^	1.895^***^	1.546^***^
1990 to 1999	(0.362)	(0.774)	(0.659)	(1.019)	(0.380)	(0.556)
Female coefficient	1.171^***^	1.049^**^	1.304^***^	2.848^***^	0.841^*^	1.538^***^
2000 to 2009	(0.317)	(0.428)	(0.446)	(0.786)	(0.479)	(0.502)
Female coefficient	1.301^***^	1.279^***^	1.989^***^	2.745^***^	1.968^***^	1.744^***^
2010 to 2019	(0.348)	(0.422)	(0.323)	(0.579)	(0.352)	(0.384)
N	782,027	732,086	410,134	395,788	697,954	699,602
Pseudo R-squared	0.331	0.335	0.376	0.441	0.278	0.294

Notes: Table entries are logistic regression coefficients: Models are fit to the set of active psychologists, economists, and mathematicians in a given year who are not yet members of the American Academy of Arts and Sciences or National Academy of Sciences. In *Panel A*′*s* parsimonious specification, we include as controls only the asinh of cumulative citations, cumulative number of publications, and year fixed effects. In *Panel B*, we add the asinh of individual journal citations, break up publications by journal, add bins of within-decade percentile citations, and add bins of decades since first publication. We also interact citation and publication variables with indicators for decade ranges (1960 to 1979, 1980 to 1999, and 2000 to 2019) to allow effects of these variables to vary over time. Standard errors, clustered by author, in parentheses. ***P < 0.01, **P < 0.05, *P < 0.1.

### Psychology.

**Fig. 2.**
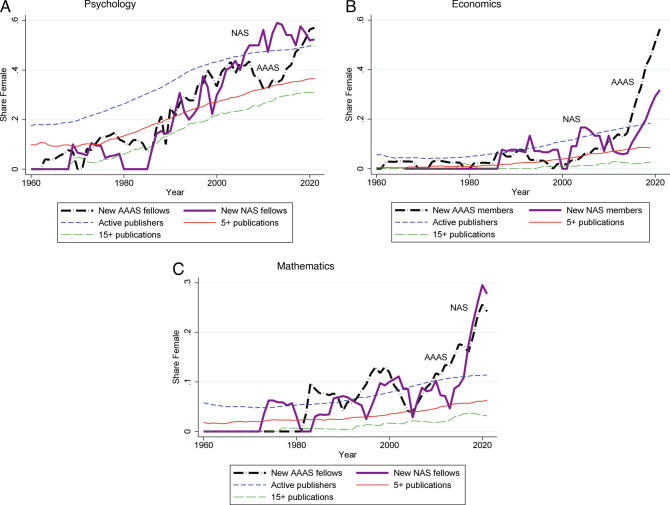
Share female of new members and active publishers. *Notes.* In this figure, we plot the share female among new members in AAAS and NAS, all active publishers, publishers with five or more publications, and publishers with 15 or more publications. The new members series excludes researchers that are not matched to the active publisher data set. AAAS and NAS shares are a moving average that include two leads, the index year, and four lags. In 2020 and 2021, we use, respectively, 1 and 0 leads. Female shares for publishers are normal averages.

We start with the field with the highest female representation, psychology. Fig. 2*A* shows the female share among the newly elected members to the AAAS and NAS. We plot 7-y moving averages to smooth out year-to-year fluctuations arising from the small number of members elected in any given year. For both academies, we see a clear increase in female representation starting from the 1980s. The figure also plots the female share in the overall sample of active publishers in psychology as well as in the sample of active publishers with at least 5 or at least 15 articles published in the high-impact journals as of year *t*. We notice two key patterns. First, all the three lines display steady, if slow, increases over time, confirming that the female share in the pipeline of potential academy members has expanded. Second, the share of females is 10 to 15% points (ppts.) lower among researchers with at least 5 publications than in the overall population of active researchers and another 5 to 7 ppts. lower among researchers with 15+ publications, implying that on average, female researchers have fewer publications than males.

When we consider the earliest three decades, 1960 to 1989, the female share of newly elected members to both AAAS and NAS is low, hovering around 5 to 15%. At the same time, the female share in the pipeline of researchers with 15+ publications is also relatively low, rising to around 15% by 1989. Overall, in this period, it appears that the female share of newly selected academy members was roughly in line with the female share of highly qualified candidates.

After 1990, the female share of new members of both academies rises from about 20% to over 40% by 2005. While there is a corresponding rise in the female share in the pipeline, the rise is slower, and even by 2020, only about 30% of researchers with 15+ publications are female. Thus, in this later period, and especially in the more recent decade, the female share among newly elected members lies substantially above the female share among highly qualified researchers, pointing to a preference for the election for female members. How does this pattern compare to other fields?

### Economics.

Next, we turn to economics, a social science with historically low shares of female researchers (21). Until the 1990s, the female share of new members of the AAAS was around 3%, while the female share of new members of the NAS was even lower. (In fact, the first female member of the NAS in economics was selected only in 1989). At the same time, the female share of researchers with 15+ publications was also very low. Between 1990 and 2010, the female share of new members at the AAAS and NAS rose to 5% and 9%, respectively. The female share of highly published authors was also rising in this period but remained below 2%. Around 2010, there was an apparent trend break in the female share of new members of both academies, with the fraction reaching 30% for the NAS and 55% for the AAAS. Was this trend break caused by a large increase in the pipeline? Fig. 2*B* shows that this is not the case, as the female share of authors with 15+ publications stays below 5% even in the most recent years. Thus, the sharp upward trend in female representation among new members of both academies in economics represents a change in the election preferences of these academies, a point we return to below.

### Mathematics.

Finally, we turn to mathematics, a science with historically low female shares of scholars. As Fig. 2*C* shows, there were essentially no female members of either academy in mathematics until 1980. This very low share is potentially explained by the very low share (< 1%) of women among researchers with 15+ publications. After 1980, the female share of newly selected academy members rose slowly from about 5% to about 10% in 2010. Over the same interval, the female share of highly published authors was also rising, albeit very slowly, reaching only about 2% in 2010. In the most recent years, the female share among the newly elected members rises above 20% for both NAS and AAAS, with a sharp increase that parallels the one in economics, though with smaller magnitudes. As is the case in economics, this trend break appears to reflect a change in the probability of selection conditional on qualifications, as opposed to a change in the pipeline, given that the female share pipeline is growing slowly over time.

A concern with the simple analysis in Fig. 2*A*–*C* is that pipelines based on publication counts underrepresent scholars who have fewer but higher impact papers. A simple alternative is to rank scholars by cumulative citations. To implement this, we created percentiles of cumulative citations to articles in the top journals, and calculated the female share among researchers in the top quarter and top 5% of citations. As a point of reference, newly elected academy members are typically in the 95th to 99th percentile of citations for their fields. *SI Appendix*, Fig. A1 *A*–*C* shows that the trends in the female share of scholars in the pipeline based on citation ranks are very similar to the shares based on publications.

A deeper concern is that simple measures of the pipeline of potential academy members based on total numbers of publications (or total numbers of citations) are flawed because different journals are accorded different weights in assessing the contributions of scholars—weights that potentially change over time. Moreover, it may be important to simultaneously control for the numbers of publications in different journals, the number of citations to articles in different journals, and other factors like the number of years that a scholar has been active. We thus turn to the regression analysis which allows for different weights on different publications, and for weights that change over time, as well as additional controls.

### Hazard Model.

We estimate a logistic hazard model for the event of being selected as a member of one of the two academies. In year *t*, the risk set includes all researchers with at least one publication in the set of top journals for a given field who are not yet members of that academy. The dependent variable is an indicator for being selected as a member in year *t*. The control variables are various measures of publications and citations as well as year effects that control for differences in the probability of selection in different years. The coefficients of interest are those associated with an indicator for female gender, which we allow to have separate effects in different decades: 1960 to 1979 (pooled to reflect the smaller number of events in these years), 1980 to 1989, 1990 to 1999, 2000 to 2009, and 2010 to 2019. Given the very low rate of selection in any year (typically around 0.1%), the point estimate for a given decade can be interpreted as the difference in the log of the probability of selection in that decade for a female candidate relative to a male candidate with similar publications and citations. A positive estimate implies a (relatively) higher selection probability for female researchers, while a negative estimate indicates a lower selection probability.

Table tab:reg presents two specifications for each field. In *Panel A*, we show our simpler specification, which has only two control variables (apart from year effects): the total number of articles that a researcher has published in the top journals of her field up to the current year and the inverse hyperbolic sine (asinh) of her total citations to previous articles in the top journals. We adopt the asinh transformation for citations, which approximates the natural log for large values of the argument, but is defined at 0. We also restrict the coefficients on the publication and citation measures to be constant across all 60 y of our sample. We view this very parsimonious specification as a proof of concept that selection as a member of either academy is strongly correlated with publications and citations and provide a much richer specification in *Panel B*.

As shown by the coefficient estimates in *Panel A*, our measure of cumulative citations is strongly correlated with induction into the AAAS and NAS, with a coefficient close to 1 for all three fields and both academies. For example, in psychology, for AAAS elections, the coefficient is 0.849 (SE = 0.093). The estimates suggest that the probability of selection is approximately unit elastic with respect to citations in the top journals for a field. Conditioning on citations, the impact of a greater number of publications is quantitatively smaller, for example, 0.045 (SE = 0.009) in psychology for AAAS. Remarkably, with just these two controls and the gender indicators, the pseudo R-squared of the models ranges between 0.20 and 0.34, indicating that citations and publications are highly predictive of selection.

The patterns of the estimated gender coefficients in *Panel A* parallel the graphical findings in Fig. 2. For the earliest time period, 1960 to 1979, we estimate negative or small positive coefficients, though we can never reject the null of a zero difference in the probability of selection for females relative to males with the same record. It thus appears that in this period, female candidates faced roughly similar or higher bars for selection as members of the two academies compared to males. We emphasize that this apparent “gender neutrality” should be interpreted in the light of the fact that women selected for honors in these years likely faced many obstacles over their careers and that no women were selected for the NAS in mathematics until 1975 and in economics until 1989.

From the 1990s onward, the estimated female coefficients are all positive, and in the last two decades, they become larger and often statistically significant. For example, for the 2010 to 2019 period, the coefficients for NAS are 1.056 (SE = 0.364) for psychology, 1.897 (SE = 0.518) for economics, and 1.173 (SE = 0.371) for mathematics. The latter coefficient implies that in mathematics, a female candidate is exp(1.17)=3.22 times more likely to be elected than a male with the same publication and citation record. This is consistent with the much higher share of females among newly elected mathematicians to the NAS in the last decade of our sample than in the pipeline of scholars with 15+ publications.

The very simple models in *Panel A* restrict the effects of citations and publications in different journals to be the same and also to be constant across decades. In *Panel B*, we present our benchmark specification which relaxes these restrictions and includes separate coefficients on publications and citations for each journal that are allowed to vary by a two-decade time period. In addition, it includes additional controls for an author’s total citation percentile (in five intervals) interacted with the time period and controls for the number of years since the author’s first publication (in four intervals). This more flexible model leads to notable gains in the pseudo R-squared coefficients, which range from 0.278 (AAAS mathematics) to 0.441 (NAS economics). We report all the coefficients in *SI Appendix*, Table S6.

The addition of the extra control variables does not change the qualitative patterns of the estimated female effects, but it tends to make the estimated female effects more positive, especially for the years after 2000. For example, the estimate for the years 2010 to 2019 for AAAS goes from 0.419 (SE = 0.290) to 1.301 (SE = 0.348) for psychology, from 1.928 (SE = 0.276) to 1.989 (SE = 0.323) for economics, and from 1.224 (SE = 0.320) to 1.968 (SE = 0.352) for mathematics. The fact that the models with additional control variables yield larger (more positive) coefficients for the female variable suggests that other unobserved quality components are most likely to be biasing these estimates downward; that is, to the extent that the unobservable components of research productivity are positively correlated with the observables measures, we would expect that if we were able to add those to the regression, the female coefficient would likely move in the same positive direction ((22)). For the earliest period, 1960 to 1979, the female coefficients remain broadly negative, if not statistically different from zero. In the 1980 to 1989 decade, only the coefficients for psychology are still negative, with economics and mathematics having quite positive coefficients. The coefficients from the 1990s on are all positive and large and statistically significant in all three fields from the 2000s on.

### Magnitudes.

**Fig. 3.**
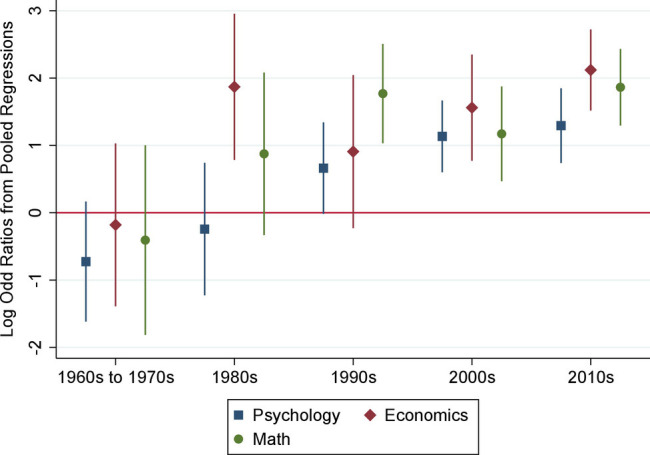
Estimated female effects in the pooled model for selection to AAAS and NAS. *Notes.* In this figure, we plot the estimated female gender coefficients from logistic regression models for the probability of being selected as a new member of the NAS or AAAS from a pooled sample, conditional on cumulative numbers of publications in top journals in the field of interest, and cumulative citations to those publications. Since the probabilities of selection as a new member are very low, these coefficients can be interpreted as effects of female gender on the log of the probability of selection. The error bars indicate the 95% CI. Regression results are presented in *SI Appendix*, Table S8.

In order to help interpret the estimates from our hazard models, we present a graphical summary in Fig. 3. To construct this figure, we note first that for all three fields, we cannot reject (using a likelihood ratio test) that the female coefficient estimates in *Panel B* are the same for the AAAS and the NAS (*P* = 0.999 in psychology, *P* = 0.521 in economics, and *P* = 0.236 in mathematics). This is an interesting finding in its own right and suggests that gender differences in the selection of new fellows may be mainly driven by norms in academia, rather than by features of the nomination and election procedures at the AAAS and NAS or by idiosyncratic preferences of voting members of the two academies. Given this finding, we estimate a pooled model imposing a common female coefficient across the two academies (while allowing the control variables to have different parameters in the selection process for the AAAS and NAS).

Fig. 3 plots the estimated coefficients for the three fields over the decades (also in *SI Appendix*, Table S8), displaying some common qualitative patterns: i) across all three disciplines, the point estimates for the earliest period (1960s to 1970s) are negative; ii) the point estimates tend to become more positive over the decades; iii) the point estimates for the two most recent decades are relatively large, positive, and precisely estimated, reflecting the larger numbers of female researchers in the later years of our sample. Comparing across the three disciplines, psychology tends to have less positive coefficients than the other two fields.

We can also interpret the magnitudes. As noted earlier, the coefficients of our logistic regression models are approximately the differences in the log of the probability of election between a female and a male candidate with similar characteristics. Considering for example psychology in the 1960s to 1970s, the estimate of −0.726 indicates that a female researcher in this earlier period was exp(−0.726) = 0.48 times as likely to be elected as a male candidate, that is, half as likely, a large difference. Conversely, in the most recent period in psychology, a female researcher is exp(1.294) = 3.65 times more likely to be elected to the academies than a male researcher with similar publications and citations. In economics and mathematics, the estimates for the most recent decade are even larger: as high as exp(2.121)=8.34 times higher for a female economist to be selected, compared to a male economist with a similar record. The results from these specifications are consistent with the visual patterns in Fig. 2*A*–*C*.

Another way to interpret the magnitudes for the most recent decade is to ask: If we were to inflate the numbers of publications and the numbers of citations of all female researchers by a certain percentage, how large would the boost have to be to fully eliminate the estimated female effect? In psychology, the estimated boost to publications and citations for female researchers is 73%. In economics and mathematics, the estimated boost is even larger, at 104% in economics and 245% in mathematics. One interpretation of this boost is that it represents the adjustment needed to compensate for the difficulties that female candidates have had in publishing their work and getting cited, e.g., in psychology, a female scholar’s publications, and citations are about 73% lower than would be expected for a male who has done similar work. An alternative interpretation is that it reflects a preference of the academies to achieve higher diversity and inclusion with respect to gender composition. We return to the interpretations of the findings in the conclusion.

### Robustness.

The specifications above make a number of assumptions, and we now discuss the robustness of the results to some key ones. One question is whether the specification controls adequately for the publications and citations in the relevant journals. In *SI Appendix*, Table S7, we show results from a specification with an intermediate set of controls between those included in the simple model of *Panel A* and the benchmark model of *Panel B*. This specification, which is similar to that used in our earlier study of honors in economics (14), produces estimates that are very close to our benchmark model. Alternatively, one would ideally like to use a larger set of journals to ensure that we capture the outlets where, for example, female researchers are more likely to publish. While it was not feasible to expand the sample in this way for all fields, in ref. (14), we estimate a model using a much larger set of 36 journals, including journals that specialize in applied fields where female researchers are more highly represented. The estimates from this model for the most recent decade are very similar, suggesting that expanding the set of journals is unlikely to change the results for the most recent period.

A different concern is that our measures of accomplishments are entirely academic and neglect other areas of contribution, such as public policy. For example, two of the female AAAS new members in economics in the 1980s had relatively limited publication records but remarkable records of public service. It is possible that accounting in systematic ways for such service would affect the estimates. However, we suspect that it might not affect the change in the estimated female coefficients as much.

## Discussion and Conclusion

How does a scholar’s gender affect her probability of induction into the prestigious AAAS and NAS academies? Using a 5-step procedure, we estimate gender gaps in selection to the academies for three fields over a 60-y time horizon. Importantly, our approach allows us to control for the research productivity of potential candidates in terms of both publications and citations in high-impact journals. Our analysis focuses on two social sciences—psychology, with a relatively high share of female scholars and female members of the AAAS and NAS, and economics with relatively low shares—and mathematics, which has relatively low shares of female scholars and academy members.

While these fields span a wide range of female representation, we find much in common in the patterns we estimate. Across all three fields, for the earliest period we study, 1960 to 1979, we find suggestive evidence that female researchers were, if anything, held to a higher bar than males. Indeed, in economics, there were no female members of the NAS elected until 1989, and in mathematics, there were no female members of the AAAS elected until 1984. This pattern, the “Matilda Effect” hypothesized by ref. (10), is consistent with anecdotal evidence of unfair treatment of female researchers in this period. It is perhaps more surprising that the extent of this finding is similar for disciplines with very few female researchers like mathematics and economics as well as for a discipline with a higher share of women like psychology.

In the next time period, 1980 to 1999, we again find a fairly similar pattern across the three fields. In this period, female researchers were generally more likely to be inducted into the AAAS and NAS conditional on publications and citations. The gender differences are small in magnitude in some cases (e.g., in psychology) and fairly large in other cases (e.g., economics in the 1980s and mathematics in the 1990s).

In the most recent decades, 2000 to 2019, we find consistent evidence that female scholars in all three fields are more likely to be inducted into the AAAS and NAS than male scholars with similar records. The magnitudes of the gender gaps in the 2010 to 2019 period are especially large and imply that women are 3 or more times more likely to be made members of the two academies than males, holding constant research productivity.

So are there no differences across fields? We do find an important difference, though perhaps not in the expected direction. In psychology, the field with the larger share of female researchers, the estimated preference for female researchers since the 1990s is in fact smaller than the one we estimate in economics and mathematics, the disciplines with a lower female representation. A possible interpretation of this finding is that members of the academies may have decided to try to redress the past underrepresentation of female scholars and have aimed at election rates for new members that are similar for men and women. In fields with lower female representation, such as economics and mathematics, this requires a more sizable boost to the election probability of female candidates. Conversely, in a field with more equal representation as psychology, this does not require a large difference. These results suggest the importance of a robust pipeline of female researchers.

We caution that our estimates are subject to the criticism that female researchers may face a harder time publishing in top journals or receiving credit for their work. In fact, there is some evidence in the recent literature of such barriers. If so, women who succeed in publishing may in fact be better scholars than men with a similar record, potentially justifying a boost in their probabilities of selection as members of the academies. To the extent that the gap in true quality between female and male scholars with similar publication records and citations has been constant over time, or at least not increasing, our results imply that there have been substantial gains in the probability of recognition for the work of female scholars at the academies.

Turning to future research, we hope that the methodology we propose and implement in this paper will be used to study other fields and/or honors as well as differences other than gender among candidates. It will also be valuable to study the impact of the nomination and election procedures for the academies, with access to confidential nomination data (which we do not have). In this regard, we cannot reject that the estimated gender differences are the same in the two academies, suggesting that the exact rules of each academy may not have played as large a role as the evolution of attitudes and preferences.

## Supplementary Material

Appendix 01 (PDF)

## Data Availability

csv files, programs data have been deposited in github to be determined.
