# Supplementary material for: Gender gaps at the academies
Source: Proc Natl Acad Sci U S A. 2023 Jan 19;120(4):e2212421120. doi: 10.1073/pnas.2212421120 (PMC9942810; doi:10.1073/pnas.2212421120)
Supplement: Supplementary file 1 — Appendix 01 (PDF) [file pnas.2212421120.sapp.pdf]

Supplementary Information for

Your main manuscript title

David Card, Stefano DellaVigna, Patricia Funk and Nagore Iriberri

David Card.
E-mail: card@econ.berkeley.edu

This PDF file includes:

Supplementary text
Fig. S1
Tables S1 to S8

Supporting Information Text

Fields in AAAS and NAS

We present here additional detail on the choice of fields for Figure 1. We download all AAAS and NAS members listed on the respective websites and we categorize their fields, sometimes grouping similar fields together so as to be able to compare across AAAS and NAS. Table S1 reports the fields with the largest number of members in the two societies, including all years. We also report the results from the gendering procedure on the percent female. (We do stress that for this large sample we did not hand check the gender coding the same way that we do for the three selected fields).

Among the three largest fields are Biochemistry, Physics, and Chemistry; from these three, we choose to feature Chemistry in Figure 1. The next largest is the Medical Sciences; however, given the existence of a separate Academy of Medicine, we do not focus on this field. The next largest field is Mathematics, Applied Mathematics, and Statistics, which is one of the three fields of focus for our analysis. The next five fields are sciences, Evolution and Ecology, Engineering and Technology, Astronomy, Astrophysics, and Earth Sciences, Neurosciences, and Cellular and Developmental Biology. We feature the last of these fields in Figure 1. The next field is the first social science, Economics, one of the fields of focus, followed by History, Literature and Language Studies, and Psychological Science, the final one of the main fields of focus. Finally, one of the next two fields is Anthropology, which we feature in Figure 1. The chosen fields for Figure 1 thus feature both natural science and social science, and include fields near the top for female representation—Cellular and Developmental Biology and Psychology and Anthropology—as well as fields low in female representation—Chemistry, Economics, and Mathematics.

Additional Details on Five-Step Procedure

Step 1. Journal Selection. The first step of the procedure is the selection of high-impact journals within a field. In principle, one could rely on impact factor rankings. However, the rankings by impact factor are affected by the fact that certain sub-fields have significantly higher citations for reasons not necessarily related to journal quality. In economics, for example, the finance journals are more cited given the large number of articles and interest in the area, so multiple finance journals would be featured in the top 15 or so most cited journals, even though AAAS and NAS members do not publish in finance journals as often as implied by this ranking. We opt instead to build a list of high-impact journals with reference directly to the journals that the elected members publish in. That is, we define high-impact journals as the ones in which the elected members publish their (well-cited) work.

Formally, for all AAAS and NAS members in a given field, we search for their Google Scholar profile. If we find it, we webscrape the top 20 papers with the most citations according to Google Scholar. We keep the name of the journals in which these articles are published, and aggregate across all the members in both AAAS and NAS to create lists of counts of articles published by journal. We then rank within a field the journals by the counts of (highly-cited) articles of elected members. We note a couple features of this measure: (i) it leads to an overrepresentation of the journals that are more relevant for the most recent periods as AAAS and NAS members prior to the 1980s are less likely to have a Google Scholar page; (ii) if a member is elected in both AAAS and NAS we count their papers twice. From the list of outlets, we eliminate: (i) books, (ii) archive websites or working papers (e.g., Arxiv Preprint), (iii) conference proceedings; (iv) journals that are so broad in field coverage that would bring in authors from other fields, leading to possible false positives, such as *Science* and *Nature.* Table S2a-c reports the list of journals in order, with emphasis on the top journals that we ultimately select. We report here just the cases that are exceptions to the rule or ties that we break.

Psychology. In psychology we take the top 13 journals in the list, excluding just *MIT Press* (as a book publisher) and *Science* (given its general-purpose outlet). We do include the *Proceedings of the National Academy of Science* given that we are able to identify the articles in the relevant field from the year 2009 on. The chosen journals include some of the general interest high-impact outlets, such as *Psychological Science*, *Psychological Review*, and *Psychological Bulletin*, as well as articles that publish articles in subfields of psychology, such as *Trends in Cognitive Sciences*, *Child Development*, and *Cognition.* We stop at journal number 13 in the list given that the next journal would be the field version of the *Journal of Experimental Psychology: General*, which we already have in the sample. As a note, the *Journal of Experimental Psychology: General* early on in the sample is simply called *Journal of Experimental Psychology.* Using these 13 journals, we are able to include over 87% of the AAAS and NAS members with a google scholar page in psychology.

Economics. In economics, we take the top 14 articles in the list, excluding working paper series (the *NBER* series), *Science* (general-purpose), and book publishers (*MIT Press* and *Cambridge University Press*). In addition to these exclusions, we also do not include the *Brookings Papers on Economic Activity*, which, while a journal, has an unusual policy target, as well as the *Journal of Financial Economics*, which would be the second finance journal in the list of journals. We also break a tie between the *Journal of Monetary Economics* and the *Economic Journal* by including the former, which allows us to include a macroeconomics field journal. The final list of 14 journals includes the traditional top-5 journals, other influential general-interest outlets such as the *Review of Economics and Statistics*, as well as one field journal in each of the following fields—theory, finance, industrial organization, econometrics, public economics, and macroeconomics. Using these 14 journals, we are able to include over 97% of the AAAS and NAS members with a google scholar page in economics, indicating that the list of journals represents especially well the most relevant outlets for members in economics.

Mathematics. In mathematics, we take the top 16 articles in the list, excluding book series (from the *American Mathematical Society, Cambridge University Press,* and *Springer*), *Science* and *Nature* (general-purpose), and conference proceedings (*IEEE Transactions On Information Theory*). We break a near-tie in favor of *Advances in Mathematics* over the *Journal of Functional*

Analysis. The 16 outlets cover journals in pure mathematics—e.g., *Advances in Mathematics* and *American Journal of Mathematics*—, journals in probability and statistics—e.g., *Annals of Probability* and the *Journal of the American Statistical Association*—, journals in applied mathematics and mathematical physics, such as *Communications on Pure and Applied Mathematics* and *Communications in Mathematical Physics*, as well as the *Proceedings of the National Academy of Sciences*. For this latter outlet, we were able to download papers in the field of mathematics from 1915-1965 and from 1996 onwards. *Annals of Probability* and *Annals of Statistics* split off from the *Annals of Mathematical Statistics* in 1972. We combine publications in *Annals of Mathematical Statistics* and *Annals of Probability*. Using these 16 journals, we are able to include over 83% of the AAAS and NAS members with a google scholar page in mathematics.

Step 2. Article and Citation Download. For all the journals that we identified as high-impact in a given field, we download the title, authors, page numbers, volume, and issue number from the earliest publication date until 2019. Specific to a given journal, we use the page number, volume, and issue number to do multiple checks that, to the extent that we can, we have all the relevant articles published by the journal with no gaps. For example, in the Quarterly Journal of Economics, the last article of Volume 137 Issue 1 ends on page 678 and the first article of Issue 2 starts on page 679, indicating the absence of gaps. We then proceed to select the relevant articles, by eliminating notes and comments which we can identify by filtering out articles containing specific words such as "note on" and "review" and "comment" in their title. Further, we additionally eliminate articles that are one or two pages in length, given that these overwhelmingly are not actual articles (e.g. obituaries), or typically make a more limited scholarly contribution. We keep articles published since 1930 in the sample, to allow for at least a 30-year record of publications for the earliest members studied in our sample (which examines member election since 1960).

For each of these articles, we locate the Web of Science citations accrued to that article in each year subsequent to the publication. For example, for an article published in 1961, we download Web of Science citations from any other journal in the Web of Science in 1961, 1962, 1963, etc. We download citations using a mix of either *doi* record or metadata of the paper (title, journal, year, volume, and page number).

Step 3. Creating Synthetic CVs. From the set of articles, we create multiple records for each article for each of the coauthors. We then merge together articles within a field written by the same author to create a data set of active publishers in a field. This is a critical step given that merging incorrectly two authors with a similar name will create artificially boosted publication records; conversely, if the record of one researcher is split under multiple records due to different spelling of a name, we will be underestimating the publications of that author, and artificially creating too many author records. Recognizing that it is impossible to achieve zero errors in this process with such a large sample, we nonetheless put in place a detailed procedure and a number of checks aimed at minimizing both types of errors.

The procedure has a number of steps aimed at connecting records with the same author name. The general rule is that we assume that the combination of first, middle, and last names uniquely identifies a researcher. We begin by replacing all non-English characters and accented characters with corresponding English characters. (For example, "á" is replaced with "a" and "ñ" is replaced with "n"). We then translated all names into a standardized format, capitalizing the first letter of first, middle, and last names, adding a dot after first or middle initials, and dropping all suffixes (including "Jr."). For example, "Trent W Appelbe" is changed to "Trent W. Appelbe". We also interchange the middle name and a leading first initial if the middle name is not an initial, e.g., "J. Bradford DeLong" is converted to "Bradford J. DeLong".

Two main complications are as follows: 1. *(alternate spelling and name formats)* an author's name may appear in slightly different ways in different articles (e.g., "Ted Bergstrom" versus "Theodore Bergstrom"); 2. *(incomplete names)* some journals, especially before 1980, identify authors only by their first initial(s) and last name (e.g., "K. Binmore" versus "Kenneth G. Binmore"). Both issues create multiple "names" for the same researcher, leading to duplicate, and thus incomplete, publication records. They also complicate our gender coding procedure (described below) which relies in part on first names.

In all cases in the following steps, we only combine names if there is only one candidate. We begin by combining names with 4 or more initials that share the same initials and last name (e.g. Douglas Allen Lauriston Auld and Douglas A. L. Auld). Next, we combine names that have the same first name and last name but contain some amount of middle initial information. This combines cases like Amartya Kumar Sen and Amartya K. Sen but not yet Amartya Sen. We then perform the same procedure for names with the same middle and last name. This combines A. Kumar Sen with Amartya Kumar Sen. We finish combining three initial names by combining names with only first and middle initial to more complete names (e.g. A. K. Sen and Amartya Kumar Sen). We then attempt to combine names containing no middle name information to a name with a longer name (e.g. A. Sen and Amartya Kumar Sen). Because of the higher error rate in this step, we additionally require that the two names have average publication years within 40 years of each other.

Following this automated name-combination procedure, we constructed a list of members and high-publishing authors for a team of research assistants to hand-check names, and combine aliases and mispellings. While checking these individuals, the research assistants also double-checked their genders along with a set of authors with uncertain genders (see Step 4). Specifically, we hand-checked all researchers with at least 1 publications in economics, at least 2 publications in psychology, and at least 3 publications in mathematics. The higher bar for hand-checks in psychology and economics is due to the larger number of individuals that are not gendered by the above procedure. A team of undergraduate research assistants checked the list of author names, looking for common short forms of names (e.g., "Larry" versus "Lawrence") and likely spelling mistakes or errors in the journal metadata. After checking these names and genders, we attempt to combine names and records again

as above. This provided a full first name for many listed authors with only a first initial, and allowed us to correct many misspelled first names.

After creating these unique researcher records, we build our main data set of active publishers in the three fields of focus. In each field, for each researcher we create annual observations for each year starting with their first publication, up to 18 years since their last publication. In addition, we remove individuals if we locate a year of death in Wikipedia. Regarding the latter check, we query all records on Wikipedia with related titles "economist," "econometrician," "anthropologist," "cognitive scientist," "linguist," "neuroscientist," "psychiatrist," "psychoanalyst," "psychologist," "psychotherapist," "social psychologist," "sociologist," "logician", "mathematician," and "statistician," as a descriptor, and collect the year of death and gender if these variables exist. We match the records to authors in the dataset using a process identical to that of author publication matching. Matched records are further reviewed by hand, and misassociated records cleaned. For the remaining matches, results from the gendering process are verified against genders provided by Wikipedia, and year of death is added to the author's profile in the dataset. Since not every author is matched to a Wikipedia page, an additional round of year of death lookups is done manually to fill in potential gaps. Research assistants additionally checked deaths during the name-checking step.

Step 4. Assigning Gender. To assign gender, we follow a procedure similar to the one outlined in Card et al. (2020). Our protocol begins by assigning "unknown gender" to common Chinese first names, since these names can be used by both males and females, and there are often multiple authors with the same Chinese name. We then use public lists of given names to idenitfy names that with high likelihood are either male or female. Specifically, we use the US and German datasets from the R package "gender". The US dataset was generated from a combination of the Social Security Administration "Baby Names" dataset. The dataset contains a list of first names and how many times the name was assigned to each gender from 1880 to 2012. The US dataset is also augmented by the US Census Data (via IPUMS). The German dataset comes from the North Atlantic Population Project and features names from Canada, Great Britain, Germany, Iceland, Norway, and Sweden.* Each of these data sets provides the frequency with which a given name is associated with a male individual.

We then assign a gender using a rule that recognizes the different Bayes rate of prevalence of females in the different disciplines, and thus a different error rate in categorization. In economics and mathematics, we classify an author as **female** if *both* the US and German lists report that less than 1% of people with that first name are male, or if the full name is present in one of the lists of female economists or mathematicians. Likewise, we classify an author as **male** if one of the US or German names lists shows that over 99% of people with that name are male and the other shows at least 50% are male. In the case that one database shows that a name is over 99% male but is missing in the other, we gender that individual as male. For females, as explained above, we use a higher threshold of consistency to avoid a high rate of false positives assigned a female gender incorrectly. For psychologists, given the higher prevalence of females in the field, we classify as **female** if less than 1% are male in one database, and the other is less than 5% or missing. In psychology we use the same classification for males as in economics and mathematics. The chosen thresholds arrive from audits of over a thousand names in different fields, using both sources like Wikipedia and hand-checks, to validate this choice of thresholds by field, and specifically ensure that the rule we choose identifies gender correctly at least 90% of the time.

This first pass still leaves a share of authors ungendered. A team of research assistants gendered as many as possible of the researchers, prioritizing researchers with more publications and Academy members. In addition to names left ungendered, the research assistants were tasked with also auditing names in cases other than when both datasets agreed on a gender with 99% accuracy. The research assistants used a combination of Wikipedia pages, researcher websites, and other online information. The team of assistants additionally attempted to combine Academy members and well-published researchers with their other records. Assistants were instructed to give extra care to names where multiple individuals could have been combined into a single record. We separate these records whenever possible.

Step 5. Match to AAAS and NAS Members. In step 5, we match the data set to a list of members of the AAAS and of the NAS in the three fields of interest. For the AAAS we identify the members as those listed on the AAAS website with specialty "Economics," "Psychology," and "Mathematics, Applied Mathematics, and Statistics." Since this list misses a number of especially the early members, we cross-check it with the AAAS Book of Members, as we now explain in detail.

We obtain a first list of respectively 438, 291, and 410 members of the AAAS from the online membership directories listed with specialty "Economics," "Psychology," and "Mathematics, Applied Mathematics, and Statistics." This list, however, does not include many members from the earlier years. We thus supplement it with information from the AAAS Book of Members and include respectively 99, 192, and 206 members elected since 1931 who are listed as economists, psychologists, and mathematicians in this additional source and are missing in the online directory (specialty "Economics", "Psychology / Education", and "Mathematics and Statistics"). There are cases where the AAAS online membership directory and the Book of Members disagree because economics, psychology, or mathematics is a secondary field (e.g., Herbert A. Simon in Economics, Gilberto Freyre in Psychology, and Michael Sipser in Mathematics). In the case of disagreement: up to and including the year 1999, we give more weight to the Book of Members and keep these members listed as primary economics, psychology or mathematics if they are listed as such in the Book of Members. From 2000 onwards, we give more weight to the online membership and follow its classification. Counting just members elected from 1960, this results in respectively 432, 251, and 400 members in Economics, Psychology, and Mathematics.

In this data set of active publishers, the first 5 female AAAS members are (i) in psychology Anne Roe (1965), Eleanor Maccoby (1974), Barbel Inhelder (1976), Eleanor Gibson (1977), and Bernice Neugarten (1980); (ii) in economics Joan Robinson

*The package infers state-recorded gender categories using historical datasets. See https://cran.r-project.org/web/packages/gender/index.html for exact details.

(1964), Irma Adelman (1974), Anne Krueger (1983), Juanita Kreps (1988), and Marina Whitman (1988); (iii) in mathematics Cathleen Morawetz (1984), Yvonne Choquet-Bruhat (1985), Julia Robinson (1985), Karen Uhlenbeck (1985), and Mary Rudin (1991). The first 5 female NAS members are (i) in psychology Eleanor Gibson (1971), Dorothea Jameson (1975), Frances Graham (1988), Barbara Partee (1989), and Eleanor Maccoby (1993); (ii) in economics Ester Boserup (1989), Anne Krueger (1995), Nancy Stokey (2004), Claudia Goldin (2006), and Susan Athey (2012); (iii) in mathematics Gertrude Cox (1975), Julia Robinson (1976), Karen Uhlenbeck (1986), Cathleen Morawetz (1990), and Marina Ratner (1993).

We note the following special cases of members that are unmatched either because they do not have any publication in the high-impact journals, or they have a publication only after they are made members. In psychology, 19 elected AAAS members are not matched with a publication in our dataset: Peter Dews (1961), Allison Daavis (1967), Charles Curle (1967), Philip Converse (1969), Daniel Lehrman (1971), Bruno Bettelheim (1971), Ralph Tyler (1976), Herman Howe Fussler (1976), Torsten Husen (1982), Vladimir Zinchenko (1989), Norma Graham (1993), Barbara Smuts (1993), Norman Bradburn (1994), Daniel Yaneklovich (1999), Alan Dundes (2001), Robert Boruch (2002), Martin Trow (2003), Roderick Little (2010), and Richard Murnane (2013). 7 members are elected more than 18 years after their last publication: Daniel Katz (1989), James House (1997), Robert Boruch (2002), Stephen Fienberg (2007), Dan Slobin (2009), William Damon (2014), and Jacques Yates (2017).

In economics, 22 elected AAAS members are not matched with a publication in our dataset: David Bell (1962), Kermit Gordon (1963), Edgar C. Brown (1966), Solomon Fabricant (1966), Barbara Ward (1966), Howard Raiffa (1968) Leonid Kantorovich (1969), Henry Rosovsky (1969), Ansley Johnson Coale (1970), Vivian Henderson (1971), Raul Prebisch (1971), Michael Postan (1972), Alfred Sauvy (1973), Kazushi Ohkawa (1973), Hans Landsberg (1974), John Crawford (1977), Emery N. Castle (1977), Edward Schuh (1977), Carlo Cipolla (1978), William Parker (1987), Harold Kuhn (1992), and Boris Saltykov (1999). 14 members are elected more than 18 years after their last publication: James Douglas Brown (1960), Lloyd Metzler (1973), Alexander Cairncross (1974), Walter Isard (1975), Eli Ginzberg (1977), Marion Clawson (1978), Duncan Black (1980), James Morgan (1984), Carlos Diaz-Alejandro (1985), Robin Matthews (1985), Alain Enthoven (1986), Marshall Goldman (1991), Robert Lampman (1991), and Cynthia Morris (1994).

In mathematics, 23 elected AAAS members are not matched with a publication in our dataset: Nikolai Bogolubov (1960), Mstislav Keldysh (1966), J. Barkley Rosser (1967), Harvey Greenspan (1968), Mark Krein (1970), John Miles (1973), Alan Perlis (1974), Sir D. Cox (1974), Igor Shafarevitch (1974), Alar Toomre (1974), Edsger Dijkstra (1975), Gabor Szego (1976), Lucien Cam (1976), Derek Moore (1985), Jacques Lions (1986), Mark Vishik (1990), Clifford Truesdelli (1991), Grigoriy Margulis (1992), Fan Graham (1998), David Crighton (1998), Anatoli Skorokhod (2000), Andrew Yao (2000), and Margaret Wright (2001). 25 members are elected more than 18 years after their last publication: Alonzo Church (1960), Heinz Hopf (1961), George Polya (1974), Kosaku Yosida (1977), Herman Wold (1978), Herman Goldstine (1983), Julia Robinson (1985), Albert tucker (1987), Alan Hoffman (1987), David Slepian (1990), Soloman Feferman (1990), Marcel-Paul Schutzenberger (1993), John Nash (1995), Elwyn Berlekamp (1996), Victor Klee (1997), John Thompson (1998), Richard Light (1998), Olga Ladyzhenskaya (2001), Joseph Harris (2002), Martin Golubitsky (2006), James Simons (2008), Louis Monvel (2012), and Michael Broue (2014).

For the NAS we include members listed on the NAS website with the primary field in the area of interest, that is, “Economic Sciences”, “Psychological and Cognitive Sciences”, and for mathematics, the two fields “Mathematics” and “Applied Mathematical Sciences”. We have 136 members with a primary field in psychology since 1960. 9 members are unmatched: Daniel Lehrman (1970), Irving Diamond (1982), Morris Halle (1988), Marianne Frankenhaeuser (1989), Mathew Alpern (1991), William Labov (1993), Norma Van Surdam Graham (1998), Edward Adelson (2006), and David Williams (2014). 4 members are elected more than 18 years after their last publication: Jack Nachmias (1984), Jozef Zwislocki (1990), Austin Riesen (1995), and Martin Banks (2019).

In economics, we include 114 members since 1960 with a primary field in Economic Sciences. Only 1 member is unmatched: Brian Berry (1975). 4 members are elected more than 18 years after their last publication: Walter Isard (1985), Hirofumi Uzawa (1995), Jacob Mincer (2000), and Allan Gibbard (2009).

In mathematics, we have 303 members with primary field in mathematics since 1960. 16 members are unmatched: A. Kolmogorov (1967), H. Longuet-Higgins (1968), Hans Liepmann (1971), I. Segal (1973), Elias Stein (1974), John Miles (1979), Mark Krein (1981), Herbert Hauptman (1988), M. Gromov (1989), John Cocke (1993), Iakov Sinai (1999), Michael Powell (2001), Margaret Wright (2005), Conjeeveram Seshadri (2010), Guinevere Kauffman (2012), and Constantine Dafermos (2016). 18 members are elected more than 18 years after their last publication: Jean Leray (1965), George Dantzig (1971), George Polya (1976), Alonzo Church (1978), Robert Hanson (1979), Alan Joffman (1982), Loo-Keng Hua (1982), David Gale (1983), Mikio Sato (1993), Maurice Stevenson Bartlett (1993), Ennio De Giorgi (1995), John Nash (1996), Edward Nelson (1997), Elwyn Berlekamp (1999), John Kingman (2007), Joseph Harris (2011), James Simons (2008), and Shigefumi Mori (2017).

Additional Details on Active Publisher Sample and Regressions

We estimate our logistic regression models for new membership from 1960 on. In the sample in year t are all researchers with at least one publication in one of the high-impact journals in that field in one of the previous 18 years, so long as they have not been elected members yet. Even though we do not run the models for years before 1960, we do record the researchers made members between 1930 and 1959 to exclude them from the regression sample.

A special case is one in which an individual has a publication in the sample of high-impact journals, but he or she is made a member before the first such publication in the sample. For example, suppose an individual is elected member in 1980, and the first publication is in 1983. In this case, the individual is in the sample in the year of election, with a record of zero

publications up to that year. Instead, if an individual is made a member more than 18 years after the last publication, that observation is not in the sample; we list all the (rare) instances of this latter case above.

Given that the AAAS and NAS memberships are not given posthumously, we aim to exclude individuals from the sample used to estimate the probability of new membership in all years after their recorded death. So if an individual has their last publication in 1985 and is recorded as having died in 1990, we remove him or her from the sample from 1991 on.

Turning to the benchmark regressions in Panel B of Table 2, the full set of coefficient estimates are in Appendix Table S6a-c. The specifications include: (i) the number of journal articles published up to that year t in each of the high-impact journals; (ii) the asinh of the total number of cumulative citations accumulated up to year t; (iii) the asinh of the number of cumulative citations for papers published in each of the high-impact journals; (iv) indicator variables for being 10-19 years from the first publications, 20-29 years from the first publications, and 30+ years from the first publication; (v) indicator variables for the individual being in a particular percentile citation group, relative to the sample of other active publishers in that field, in that decade, where we take the relevant percentiles to be 90th-95th, 95th-99th, 99th-99.5th, and 99.5th+; (vi) year fixed effects.

Appendix Figure S1. Share Female of New Members and Publishers

Panel A. Psychology

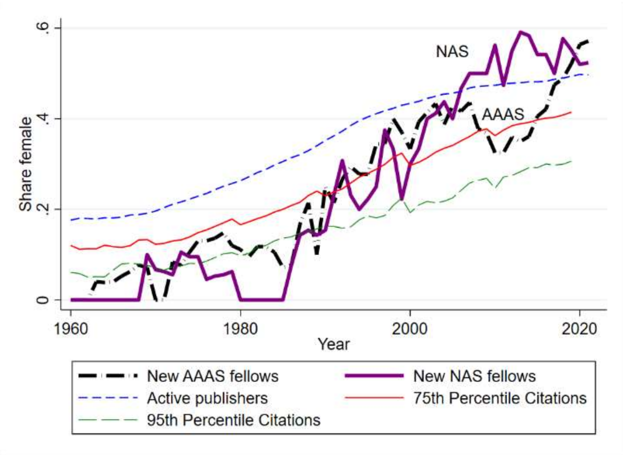

Panel B. Economics

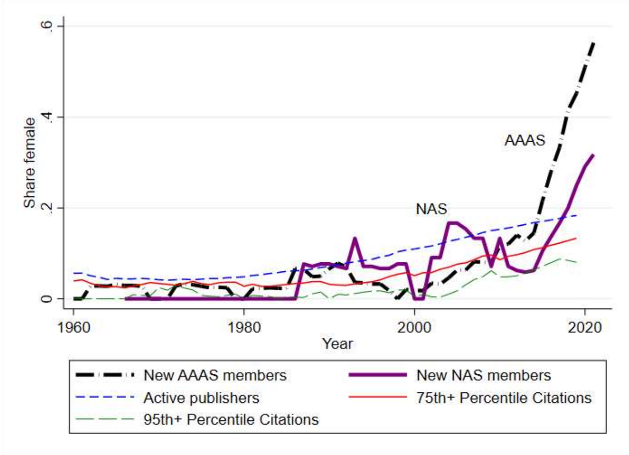

Panel C. Mathematics

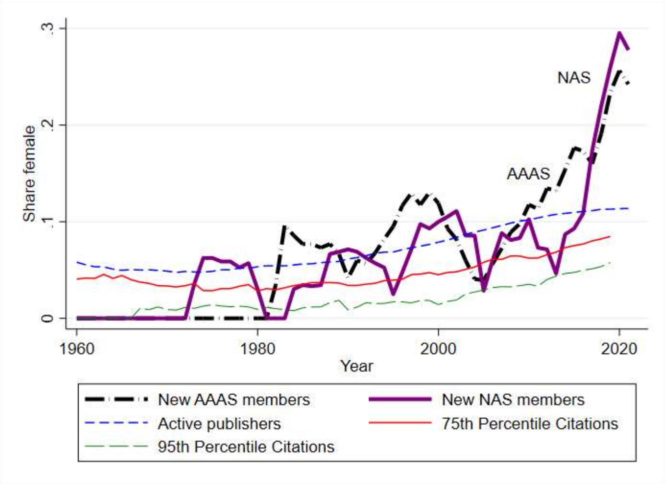

Notes. In this figure, we plot the share female for 5 samples in each field: (1) new members of AAAS who have publications in our database; (2) new members of NAS who have publications in our database; (3) all active publishers; (4) active publishers with cumulative citations as of the given year at the 75th percentile or above; (5) active publishers with cumulative citations as of the given year at the 90th percentile or above. AAAS and NAS shares are a moving average that include 2 leads, the index year, and 4 lags. In 2020 and 2021, we use respectively 1 and 0 leads. Percentiles of citations are computed within-decade.

APPENDIX TABLE S1 MAJOR FIELDS IN AAAS AND NAS

	Total Members	AAAS Members	NAS Members	Percent Female
Biochemistry, Biophysics, and Molecular Biology	950	541	409	12.96%
Physics	874	498	376	5.64%
Chemistry	843	448	395	7.63%
Medical Sciences	783	473	310	12.31%
Mathematics, Applied Mathematics, and Statistics	651	410	241	6.18%
Evolution and Ecology	604	394	210	17.94%
Engineering and Technology	581	394	187	8.48%
Astronomy, Astrophysics, and Earth Sciences	577	418	159	11.78%
Neurosciences	571	365	206	16.52%
Cellular and Developmental Biology	565	379	186	19.68%
Economics	557	438	119	7.36%
History	519	519	0	16.73%
Literature and Language Studies	443	443	0	13.79%
Psychological Sciences	442	291	151	22.95%
Visual Arts	415	415	0	16.58%
Anthropology and Archaeology	384	223	161	21.47%
Law	368	368	0	13.48%

Notes. In this table, we show the total number of members and the percentage of females in the AAAS and NAS. To make the AAAS and NAS more comparable, we combine the following AAAS fields: "Biochemistry" and "Biophysics and Computational Biology" to "Biochemistry, Biophysics, and Molecular Biology", "Environmental Sciences and Ecology" and "Evolutionary Biology" to "Evolution and Ecology", "Medical Genetics, Hematology, and Oncology" to "Medical Physiology and Metabolism", "Microbial Biology" and "Immunology and Inflamation" and "Microbiology and Immunology", and "Cellular and Molecular Neuroscience" and "Systems Neuroscience" to "Neurosciences". There is no corresponding field for "History," "Literature and Language Studies," "Visual Arts," or "Law" in the NAS. In Psychology, Economics, and Mathematics, we use the best possible gendering that include hand-checks. We gender algorithmically in other fields. The percentage excludes members with an unknown gender.

APPENDIX TABLE S2A JOURNAL SELECTION - PSYCHOLOGY

	No. Papers	No. Papers (AAAS Members)	No. Papers (NAS Members)
Journal Of Personality And Social Psychology	202	160	42
Psychological Review	166	104	62
Cognition	112	64	48
Science	107	63	44
Child Development	78	62	16
Cognitive Psychology	74	46	28
American Psychologist	74	53	21
Psychological Science	66	44	22
Psychological Bulletin	65	56	9
Trends In Cognitive Sciences	55	30	25
Mit Press	51	27	24
Annual Review Of Psychology	51	40	11
Journal Of Experimental Psychology: General	48	28	20
Proceedings Of National Academy Of Sciences	45	25	20
Developmental Psychology	44	35	9
Cambridge University Press	41	27	14
Journal Of Experimental Psychology: Learning, Memory, And Cognition	38	24	14
Journal Of Memory And Language	37	22	15

Notes. In this table, we show the journals in which members in the AAAS and NAS publish. For any member with a Google Scholar page, we took their 20 most-cited papers and recorded the journal where they published. Highlighted journals are included in our analyses.

APPENDIX TABLE S2B JOURNAL SELECTION - ECONOMICS

	No. Papers	No. Papers (AAAS Members)	No. Papers (NAS Members)
American Economic Review	583	447	136
Econometrica	460	349	111
Quarterly Journal Of Economics	406	330	76
Journal Of Political Economy	353	268	85
National Bureau Of Economic Research	251	197	54
Review Of Economic Studies	187	158	29
Journal Of Economic Theory	155	114	41
Journal Of Economic Perspectives	148	117	31
Journal Of Finance	107	88	19
Journal Of Economic Literature	89	68	21
Brookings Papers On Economic Activity	82	62	20
Mit Press	81	62	19
Rand Journal Of Economics	77	61	16
Journal Of Econometrics	65	54	11
Journal Of Public Economics	62	56	6
Review Of Economics And Statistics	56	43	13
Cambridge University Press	54	41	13
Science	51	39	12
Journal Of Financial Economics	51	45	6
Economic Journal	50	42	8
Journal Of Monetary Economics	50	40	10
European Economic Review	46	40	6

Notes. In this table, we show the journals in which fellows in the AAAS and NAS publish. For any Fellow with a Google Scholar page, we took their 20 most-cited papers and recorded the journal where they published. Highlighted journals are included in our analyses.

APPENDIX TABLE S2C JOURNAL SELECTION - MATHEMATICS

	No. Papers	No. Papers (AAAS Members)	No. Papers (NAS Members)
Annals Of Mathematics	153	96	57
Communications On Pure And Applied Mathematics	142	94	48
Communications In Mathematical Physics	104	65	39
Arxiv Preprint	86	48	38
American Mathematical Soc.	76	47	29
Annals Of Statistics	76	35	41
Inventiones Mathematicae	75	50	25
Proceedings Of National Academy Of Sciences	73	38	35
Journal Of American Mathematical Society	69	43	26
Duke Mathematical Journal	67	41	26
Journal Of American Statistical Association	64	33	31
Journal Of Computational Physics	61	22	39
Acta Mathematica	50	32	18
Springer Science & Business Media	48	25	23
Cambridge University Press	48	28	20
Annals Of Probability	48	31	17
Nature	46	15	31
Springer	46	25	21
Transactions Of American Mathematical Society	46	28	18
Annals Of Mathematical Statistics	44	26	18
American Journal Of Mathematics	43	28	15
Ieee Transactions On Information Theory	37	21	16
Journal Of Functional Analysis	36	32	4
Advances In Mathematics	35	19	16
Journal Of Fluid Mechanics	31	27	4

Notes. In this table, we show the journals in which members in the AAAS and NAS publish. For any member with a Google Scholar page, we took their 20 most-cited papers and recorded the journal where they published. Highlighted journals are included in our analyses.

APPENDIX TABLE S3A PSYCHOLOGY
SUMMARY STATISTICS FOR DATA SET OF ACTIVELY PUBLISHING PSYCHOLOGISTS

	**Psychologists with at Least One Paper Published in Set of Leading Journals**								
	1960-1979			1980-1999			2000-2019		
	All	Male	Female	All	Male	Female	All	Male	Female
	(1)	(2)	(3)	(4)	(5)	(6)	(7)	(8)	(9)
Percent Female	19.18	0	100	31.63	0	100	42.98	0	100
Percent Unknown First Initial Only	6	0	0	3.17	0	0	1.21	0	0
Percent Unknown Full Name	4.6	0	0	6.13	0	0	7.49	0	0
Percent Members (as of current year)									
Current Member of AAAS (1960+)	0.35	0.47	0.1	0.41	0.59	0.19	0.48	0.67	0.36
Current Member of NAS (1960+)	0.19	0.26	0.05	0.31	0.49	0.07	0.23	0.33	0.17
Cumulative publications in psychology journals									
Psychological Science (1990 -)	0	0	0	0.02	0.02	0.02	0.28	0.35	0.23
Psychological Review (1984 -)	0.16	0.2	0.05	0.09	0.14	0.03	0.1	0.16	0.03
Psychological Bulletin (1904 -)	0.18	0.22	0.07	0.17	0.24	0.07	0.12	0.18	0.07
Trends in Cognitive Sciences (1997 -)	0	0	0	0	0	0	0.09	0.14	0.05
J. of Experimental Psych: General (1975 -)	0.95	1.12	0.51	0.41	0.59	0.15	0.17	0.27	0.09
J. of Personality and Social Psychology (1965 -)	0.31	0.36	0.22	0.64	0.8	0.46	0.63	0.88	0.44
Annual Review of Psychology (1950 -)	0.05	0.05	0.02	0.05	0.06	0.02	0.05	0.07	0.03
American Psychologist (1946 -)	0.16	0.19	0.07	0.2	0.27	0.12	0.15	0.21	0.1
Child Development (1930 -)	0.35	0.32	0.57	0.49	0.45	0.64	0.48	0.45	0.57
Cognition (1972 -)	0.01	0.01	0.01	0.05	0.06	0.05	0.21	0.26	0.18
Cognitive Psychology (1970 -)	0.02	0.02	0.02	0.05	0.05	0.04	0.06	0.08	0.05
Developmental Psychology (1969 -)	0.09	0.08	0.14	0.28	0.25	0.38	0.39	0.35	0.47
PNAS (2009 -)	0	0	0	0	0	0	0.07	0.09	0.05
Cumulative citations in psychology journals									
Psychological Science (1990 -)	0	0	0	0.17	0.2	0.14	13.16	17.07	10.25
Psychological Review (1984 -)	4.45	5.76	1.32	7.76	11.89	2.06	19.52	32.69	8.24
Psychological Bulletin (1904 -)	3.87	4.88	1.5	8.86	12.65	3.56	22.23	33.46	13.19
Trends in Cognitive Sciences (1997 -)	0	0	0	0.01	0.01	0	8.9	13.8	4.7
J. of Experimental Psych: General (1975 -)	10.09	12.32	5.44	8.15	11.77	3.19	9.33	14.42	5.09
J. of Personality and Social Psychology (1965 -)	3.01	3.49	2.43	16.61	20.52	12.78	52.97	73.79	38.34
Annual Review of Psychology (1950 -)	0.37	0.38	0.15	1.54	1.79	0.68	6.98	10.04	4.34
American Psychologist (1946 -)	1.12	1.48	0.31	4.46	5.99	2.58	12.75	18.49	8.48
Child Development (1930 -)	1.15	1.11	1.72	4.21	4.07	5.22	17.33	16.38	20.59
Cognition (1972 -)	0.02	0.02	0.03	1.06	1.13	1.02	9.73	12.08	8.28
Cognitive Psychology (1970 -)	0.21	0.24	0.18	2.55	3.01	2.33	5.99	7.84	4.89
Developmental Psychology (1969 -)	0.47	0.44	0.71	4.14	3.89	5.33	17.12	16.91	19.56
PNAS (2009 -)	0	0	0	0	0	0	2.63	3.27	1.97
Number of years since first publication	8.24	8.67	6.94	11.53	12.99	9.4	11.47	13.31	10.11
Number of Author-Year Observations	148,608	104,355	28,499	305,345	180,361	96,573	435,187	210,278	187,030
Number of Authors	14,771	9,778	3,282	26,614	15,066	8,837	43,982	20,395	19,047

Notes. Data set contains author-year observations on "actively publishing" psychologists. A psychologist becomes active upon publishing a paper in one of the journals listed in Appendix Table 2a, and remains active for 18 years after the last publication. Gender is based on name or internet search -- see text. Citations in psychology journals are measured from Web of Science SSCI and are cumulative up to that year.

APPENDIX TABLE S3B ECONOMICS
SUMMARY STATISTICS FOR DATA SET OF ACTIVELY PUBLISHING Economists

	**Economists with at Least One Paper Published in Set of Leading Journals**								
	1960-1979			1980-1999			2000-2019		
	All	Male	Female	All	Male	Female	All	Male	Female
	(1)	(2)	(3)	(4)	(5)	(6)	(7)	(8)	(9)
Percent Female	4.03	0	100	7.08	0	100	14.69	0	100
Percent Unknown First Initial Only	6.59	0	0	3.06	0	0	0.6	0	0
Percent Unknown Full Name	2.22	0	0	2.35	0	0	1.47	0	0
Percent Members (as of current year)									
Current Member of AAAS (1960+)	1.83	2.04	1.11	1.86	2.06	0.73	1.94	2.22	0.64
Current Member of NAS (1960+)	0.25	0.29	0	0.55	0.62	0.1	0.5	0.57	0.19
Cumulative publications in Economics journals									
American Economic Review (1911 -)	0.44	0.47	0.3	0.38	0.41	0.2	0.4	0.44	0.25
Econometrica (1933 -)	0.35	0.37	0.12	0.36	0.39	0.1	0.33	0.37	0.09
Journal of Political Economy (1892 -)	0.43	0.47	0.21	0.32	0.35	0.14	0.25	0.28	0.11
Quarterly Journal of Economics (1886 -)	0.34	0.37	0.24	0.22	0.24	0.12	0.22	0.24	0.13
Review of Economic Studies (1933 -)	0.2	0.2	0.11	0.22	0.23	0.08	0.22	0.24	0.1
Journal of Econometrics (1973 -)	0.02	0.02	0.02	0.2	0.21	0.12	0.39	0.42	0.2
Journal of Economic Theory (1968 -)	0.07	0.08	0.03	0.25	0.27	0.11	0.38	0.43	0.14
RAND Journal of Economics (1970 -)	0.04	0.04	0.02	0.15	0.16	0.14	0.18	0.19	0.13
Journal of Economic Perspectives (1987 -)	0	0	0	0.03	0.04	0.04	0.13	0.13	0.11
Journal of Economic Literature (1963 -)	0.01	0.01	0.02	0.03	0.04	0.02	0.05	0.05	0.03
Review of Economics and Statistics (1919 -)	0.42	0.44	0.39	0.38	0.39	0.39	0.34	0.36	0.27
Journal of Finance (1946 -)	0.31	0.34	0.16	0.38	0.41	0.17	0.37	0.41	0.21
Journal of Monetary Economics (1973 -)	0.01	0.01	0.01	0.11	0.12	0.08	0.22	0.23	0.14
Journal of Public Economics (1972 -)	0.02	0.02	0.02	0.18	0.18	0.13	0.35	0.37	0.28
Cumulative citations in Economics journals									
American Economic Review (1911 -)	2.33	2.56	1.42	9.1	10.03	3.54	30.13	32.9	18.01
Econometrica (1933 -)	2.55	2.73	0.63	9.33	10.38	1.46	28.7	33.34	6.11
Journal of Political Economy (1892 -)	1.8	1.99	0.8	7.96	8.83	2.61	22.97	26.32	7.1
Quarterly Journal of Economics (1886 -)	1.1	1.19	0.88	3.23	3.53	1.53	21.05	23.21	11.64
Review of Economic Studies (1933 -)	0.71	0.74	0.45	3.12	3.42	0.7	10.17	11.58	3.5
Journal of Econometrics (1973 -)	0	0	0	1.3	1.36	0.65	11.2	12.26	4.58
Journal of Economic Theory (1968 -)	0.24	0.27	0.03	3.07	3.41	0.84	9.18	10.54	2.43
RAND Journal of Economics (1970 -)	0	0	0	0.65	0.68	0.65	5.45	5.76	4.37
Journal of Economic Perspectives (1987 -)	0	0	0	0.23	0.25	0.14	5.06	5.4	3.75
Journal of Economic Literature (1963 -)	0.1	0.11	0.08	1.13	1.26	0.39	5.79	6.42	2.83
Review of Economics and Statistics (1919 -)	1.48	1.62	0.92	3.95	4.24	2.5	10.46	10.97	8.5
Journal of Finance (1946 -)	0.56	0.62	0.12	4.17	4.62	1.07	26.89	29.91	12.92
Journal of Monetary Economics (1973 -)	0.01	0.01	0.01	1.46	1.59	0.8	7.65	8.56	3.53
Journal of Public Economics (1972 -)	0.01	0.01	0	1.15	1.24	0.69	7.75	8.17	6.19
Number of years since first publication	10.18	10.4	9.12	11.24	11.56	8.02	13.04	13.74	9.37
Number of Author-Year Observations	60,163	52,443	2,423	148,007	129,526	10,476	230,080	191,517	33,795
Number of Authors	6,314	5,468	294	12,388	10,611	1,096	20,292	16,517	3,225

Notes. Data set contains author-year observations on "actively publishing" Economists. A Economist becomes active upon publishing a paper in one of the journals listed in Appendix Table 2b, and remains active for 18 years after the last publication. Gender is based on name or internet search -- see text. Citations in economics journals are measured from Web of Science SSCI and are cumulative up to that year.

APPENDIX TABLE S3C MATHEMATICS
SUMMARY STATISTICS FOR DATA SET OF ACTIVELY PUBLISHING MATHEMATICIANS

	**Mathematicians with at Least One Paper Published in Set of Leading Journals**								
	1960-1979			1980-1999			2000-2019		
	All	Male	Female	All	Male	Female	All	Male	Female
	(1)	(2)	(3)	(4)	(5)	(6)	(7)	(8)	(9)
Percent Female	3.63	0	100	4.45	0	100	7.19	0	100
Percent Unknown First Initial Only	21.57	0	0	21.37	0	0	14.55	0	0
Percent Unknown Full Name	4.91	0	0	7.86	0	0	14.14	0	0
Percent Members (as of current year)									
Current Member of AAAS (1960+)	0.73	1.05	0	0.82	1.19	0.58	0.71	1.03	0.74
Current Member of NAS (1960+)	0.5	0.7	0.11	0.6	0.89	0.24	0.53	0.78	0.47
Cumulative publications in mathematics journals									
Advances in Mathematics (1961 -)	0.02	0.03	0.02	0.07	0.1	0.07	0.16	0.21	0.16
American Journal of Mathematics (1878 -)	0.27	0.35	0.1	0.14	0.19	0.07	0.09	0.13	0.06
Annals of Mathematics (1874 -)	0.3	0.41	0.09	0.14	0.2	0.04	0.1	0.15	0.04
Annals of Probability (1974 -)	0.03	0.03	0.02	0.14	0.18	0.11	0.15	0.2	0.11
Annals of Statistics (1973 -)	0.5	0.6	0.34	0.35	0.45	0.27	0.22	0.3	0.2
Acta Mathematica (1882 -)	0.08	0.1	0.02	0.05	0.07	0.03	0.03	0.05	0.02
Comm. on Pure and Applied Math (1948 -)	0.12	0.14	0.13	0.1	0.13	0.11	0.09	0.13	0.08
Comm. in Mathematical Physics (1965 -)	0.11	0.1	0.05	0.32	0.34	0.15	0.41	0.51	0.2
Duke Mathematical Journal (1935 -)	0.34	0.41	0.15	0.16	0.22	0.1	0.14	0.19	0.1
Inventiones Mathematicae (1966 -)	0.05	0.07	0.02	0.16	0.21	0.12	0.15	0.21	0.09
Journal of the American Math. Society (1988 -)	0	0	0	0.01	0.02	0.01	0.04	0.06	0.03
Journal of the American Stat. Assn. (1922 -)	0.33	0.38	0.43	0.3	0.37	0.4	0.26	0.32	0.39
Journal of Computational Physics (1966 -)	0.09	0.07	0.04	0.29	0.26	0.13	0.58	0.61	0.34
PNAS (1915 - 1965, 1996 -)	0.18	0.23	0.08	0.03	0.04	0.01	0.06	0.07	0.07
Transactions of the American Math. Soc. (1900 -)	0.58	0.69	0.43	0.53	0.64	0.42	0.41	0.51	0.36
Cumulative citations in mathematics journals									
Advances in Mathematics (1961 -)	0.07	0.09	0.05	0.82	1.17	0.39	2.58	3.53	1.91
American Journal of Mathematics (1878 -)	1.17	1.6	0.16	1.44	2.06	0.33	1.61	2.33	0.79
Annals of Mathematics (1874 -)	2.5	3.49	0.45	3.25	4.76	0.65	4.23	6.31	1.52
Annals of Probability (1974 -)	0.04	0.05	0.02	1	1.33	0.55	2.46	3.36	1.44
Annals of Statistics (1973 -)	3.57	4.55	1.74	4.94	6.8	2.68	7.83	11.13	5.12
Acta Mathematica (1882 -)	0.5	0.67	0.03	0.99	1.41	0.49	1.44	2.12	0.64
Comm. on Pure and Applied Math (1948 -)	0.95	1.22	0.57	2.09	2.92	1.79	4.54	6.22	5.03
Comm. in Mathematical Physics (1965 -)	0.43	0.39	0.15	5.06	5.68	1.68	11.13	14.6	3.47
Duke Mathematical Journal (1935 -)	0.91	1.14	0.34	1.15	1.58	0.46	2.45	3.49	1.48
Inventiones Mathematicae (1966 -)	0.12	0.16	0.02	1.83	2.51	0.94	4.45	6.48	2.17
Journal of the American Math. Society (1988 -)	0	0	0	0.01	0.01	0.01	0.9	1.28	0.66
Journal of the American Stat. Assn. (1922 -)	2.02	2.5	1.95	4.93	6.63	4.59	11.51	15.38	13.39
Journal of Computational Physics (1966 -)	0.14	0.11	0.05	3.11	3.1	1.08	16.94	19.97	8.33
PNAS (1915 - 1965, 1996 -)	0.68	0.89	0.36	0.28	0.4	0.14	3.4	3.9	5.53
Transactions of the American Math. Soc. (1900 -)	1.75	2.23	0.71	3.32	4.31	1.97	5.05	6.64	3.73
Number of years since first publication	9.21	9.96	9.04	11.35	12.32	9.25	12.15	13.61	10.1
Number of Author-Year Observations	129,817	90,722	4,714	311,824	206,787	13,879	555,438	356,154	39,936
Number of Authors	13,104	8,812	514	27,577	17,552	1,379	50,743	30,726	3,658

Notes. Data set contains author-year observations on "actively publishing" mathematicians. A mathematician becomes active upon publishing a paper in one of the journals listed in Appendix Table 2c, and remains active for 18 years after the last publication. Gender is based on name or internet search -- see text. Citations in mathematics journals are measured from Web of Science SSCI and are cumulative up to that year.

APPENDIX TABLE S4A PSYCHOLOGY
SUMMARY STATISTICS FOR AAAS MEMBERS IN ELECTION YEAR

	Newly Elected Members in Year of Election								
	1960-1979			1980-1999			2000-2019		
	All	Male	Female	All	Male	Female	All	Male	Female
	(1)	(2)	(3)	(4)	(5)	(6)	(7)	(8)	(9)
Percent Female	7.14	0	100	25.71	0	100	39.62	0	100
Percent Unknown Gender	0	0	0	0	0	0	0	0	0
Percent Members (as of current year)									
Current Member of AAAS (1960+)	100	100	100	100	100	100	100	100	100
Current Member of NAS (1960+)	14.29	13.46	25	14.29	17.31	5.56	3.77	3.13	4.76
Cumulative publications in psychology journals									
Psychological Science (1990 -)	0	0	0	0.17	0.19	0.11	2.48	2.58	2.33
Psychological Review (1984 -)	1.23	1.29	0.5	1.57	2.02	0.28	1.02	1.25	0.67
Psychological Bulletin (1904 -)	0.61	0.62	0.5	0.51	0.65	0.11	1.18	1.47	0.74
Trends in Cognitive Sciences (1997 -)	0	0	0	0	0	0	0.59	0.58	0.62
J. of Experimental Psych: General (1975 -)	2.21	2.21	2.25	2.27	2.85	0.61	1.14	1.31	0.88
J. of Personality and Social Psychology (1965 -)	0.75	0.75	0.75	3.09	2.92	3.56	7.91	10.33	4.21
Annual Review of Psychology (1950 -)	0.36	0.33	0.75	0.33	0.33	0.33	0.64	0.73	0.5
American Psychologist (1946 -)	0.96	0.98	0.75	1.21	1.15	1.39	1.33	1.47	1.12
Child Development (1930 -)	0.68	0.56	2.25	1.91	1.77	2.33	3.97	3.75	4.31
Cognition (1972 -)	0.02	0	0.25	0.76	0.62	1.17	2.09	1.66	2.76
Cognitive Psychology (1970 -)	0.11	0.1	0.25	0.93	1	0.72	0.83	0.67	1.07
Developmental Psychology (1969 -)	0	0	0	0.84	0.75	1.11	2.97	3.31	2.45
PNAS (1915 -)	0	0	0	0	0	0	0.23	0.28	0.14
Cumulative citations in psychology journals									
Psychological Science (1990 -)	0	0	0	2.16	2.9	0	137.75	144.27	127.83
Psychological Review (1984 -)	69.43	66.9	102.25	299.27	385.38	50.5	430.41	524.67	286.76
Psychological Bulletin (1904 -)	33.21	34.13	21.25	35.74	47.56	1.61	334.94	400.03	235.76
Trends in Cognitive Sciences (1997 -)	0	0	0	0	0	0	57.3	65.48	44.83
J. of Experimental Psych: General (1975 -)	52.05	50.08	77.75	112.41	141.54	28.28	119.98	156.41	64.48
J. of Personality and Social Psychology (1965 -)	13.77	14.4	5.5	187.71	149.27	298.78	991.31	1,245.17	604.48
Annual Review of Psychology (1950 -)	2.43	1.9	9.25	18.31	16.98	22.17	140.68	167.41	99.95
American Psychologist (1946 -)	17.45	17.04	22.75	51.93	51.48	53.22	159.24	171.34	140.79
Child Development (1930 -)	3.61	2.29	20.75	20.87	14.4	39.56	188.18	234.14	118.14
Cognition (1972 -)	0	0	0	24.99	14.96	53.94	147.74	145.33	151.4
Cognitive Psychology (1970 -)	3.16	2.79	8	102.8	91.62	135.11	111.01	126.75	87.02
Developmental Psychology (1969 -)	0	0	0	21.41	17.71	32.11	168.4	204.83	112.88
PNAS (2009 -)	0	0	0	0	0	0	10.01	13.75	4.31
Number of years since first publication	17.80	17.63	20.00	24.40	25.56	21.06	31.36	32.47	29.67
Number of Authors	56	52	4	70	52	18	106	64	42
Unmatched Members	8	7	1	6	4	2	5	5	0

Notes: Table presents characteristics of psychologists in the years of election as members of the American Academy of Arts and Sciences.

APPENDIX TABLE S4B ECONOMICS
SUMMARY STATISTICS FOR AAAS MEMBERS IN ELECTION YEAR

	Newly Elected Members in Year of Election								
	1960-1979			1980-1999			2000-2019		
	All	Male	Female	All	Male	Female	All	Male	Female
	(1)	(2)	(3)	(4)	(5)	(6)	(7)	(8)	(9)
Percent Female	1.87	0	100	3.68	0	100	15.57	0	100
Percent Unknown Gender	0	0	0	0	0	0	0	0	0
Percent Members (as of current year)									
Current Member of AAAS (1960+)	100	100	100	100	100	100	100	100	100
Current Member of NAS (1960+)	0	0	0	0.74	0.76	0	0	0	0
Cumulative publications in econ journals									
American Economic Review	1.64	1.63	2	2.01	2.07	0.6	2.3	2.39	1.81
Econometrica	1.93	1.94	1	2.83	2.93	0.2	2.97	3.24	1.5
Journal of Political Economy	1.5	1.53	0	2.01	2.04	1.2	1.85	1.99	1.12
Quarterly Journal of Economics	1.04	1.01	2.5	1.48	1.48	1.4	2.47	2.51	2.23
Review of Economic Studies	1.07	1.02	3.5	1.74	1.79	0.4	1.57	1.77	0.46
Journal of Econometrics	0.05	0.05	0	0.84	0.87	0	0.81	0.79	0.96
Journal of Economic Theory	0.18	0.18	0	1.57	1.62	0.4	1.93	2.2	0.46
RAND Journal of Economics	0.1	0.1	0	0.8	0.82	0.2	0.58	0.61	0.42
Journal of Economic Perspectives	0	0	0	0.29	0.31	0	1.07	1.13	0.73
Journal of Economic Literature	0.07	0.08	0	0.2	0.19	0.4	0.3	0.28	0.38
Review of Economics and Statistics	1.21	1.21	1	0.6	0.63	0	0.48	0.48	0.5
Journal of Finance	0.16	0.16	0	0.48	0.49	0.2	0.77	0.87	0.23
Journal of Monetary Economics	0	0	0	0.41	0.42	0.2	0.77	0.81	0.54
Journal of Public Economics	0.15	0.15	0	0.79	0.82	0	0.9	0.91	0.85
Cumulative citations in econ journals									
American Economic Review (1911 -)	14.08	13.77	30.5	87.21	90.1	11.6	210.99	208.55	224.23
Econometrica (1933 -)	19.24	19.39	11.5	142.37	147.4	10.4	278.42	308.69	114.27
Journal of Political Economy (1892 -)	9.28	9.46	0	95.35	97.92	27.8	212.73	228.44	127.54
Quarterly Journal of Economics (1886 -)	6.23	6.04	16.5	35.4	35.89	22.6	268.2	270.4	256.27
Review of Economic Studies (1933 -)	5.11	5.06	8	41.6	43.14	1.4	106.65	122.52	20.62
Journal of Econometrics (1973 -)	0	0	0	14.18	14.73	0	30.96	31.05	30.5
Journal of Economic Theory (1968 -)	1.08	1.1	0	50.62	51.74	21.2	68.16	78.48	12.23
RAND Journal of Economics (1970 -)	0	0	0	4.68	4.86	0	36.31	38.56	24.08
Journal of Economic Perspectives (1987 -)	0	0	0	1.65	1.72	0	33.25	34.79	24.88
Journal of Economic Literature (1963 -)	0.66	0.68	0	8.65	8.63	9.2	41.32	35.7	71.85
Review of Economics and Statistics (1919 -)	4.52	4.47	7.5	11.4	11.84	0	24.32	25.66	17.04
Journal of Finance (1946 -)	0.18	0.18	0	15.82	16.39	0.8	79.85	89.01	30.19
Journal of Monetary Economics (1973 -)	0	0	0	11.11	10.52	26.6	32.51	34.52	21.62
Journal of Public Economics (1972 -)	0.09	0.1	0	10.57	10.97	0	34.46	23.68	92.92
Number of years since first publication	20.78	20.7	24.5	20.9	21.17	14	22.38	22.67	20.85
Number of Authors	107	105	2	136	131	5	167	141	26
Unmatched Members	19	18	1	3	3	0	0	0	0

Notes: Table presents characteristics of economists in the years of election as Members of the American Academy of Arts and Sciences.

APPENDIX TABLE S4C MATHEMATICS
SUMMARY STATISTICS FOR AAAS MEMBERS IN ELECTION YEAR

	Newly Elected Members in Year of Election								
	1960-1979			1980-1999			2000-2019		
	All	Male	Female	All	Male	Female	All	Male	Female
	(1)	(2)	(3)	(4)	(5)	(6)	(7)	(8)	(9)
Percent Female	0	0	--	9.23	0	100	11.11	0	100
Percent Unknown Gender	0	0	--	0	0	0	0	0	0
Percent Members (as of current year)									
Current Member of AAAS (1960+)	100	100	--	100	100	100	100	100	100
Current Member of NAS (1960+)	11.65	11.65	--	21.45	24.37	0	16.67	16.41	18.75
Cumulative publications in math journals									
Advances in Mathematics (1961 -)	0.08	0.08	--	0.68	0.73	0.25	0.78	0.82	0.50
American Journal of Mathematics (1878 -)	1.91	1.91	--	0.72	0.76	0.25	0.96	0.98	0.75
Annals of Mathematics (1874 -)	3.48	3.48	--	1.42	1.47	0.83	1.73	1.82	1.00
Annals of Probability (1974 -)	0.03	0.03	--	0.52	0.58	0.00	1.05	1.14	0.31
Annals of Statistics (1973 -)	2.74	2.74	--	2.21	2.33	1.00	0.78	0.88	0.00
Acta Mathematica (1882 -)	0.41	0.41	--	0.58	0.59	0.42	0.44	0.41	0.63
Comm. on Pure and Applied Math (1948 -)	1.09	1.09	--	1.31	1.27	1.67	1.99	2.04	1.63
Comm. in Mathematical Physics (1965 -)	0.04	0.04	--	1.19	1.19	1.17	2.55	2.67	1.56
Duke Mathematical Journal (1935 -)	1.15	1.15	--	0.73	0.75	0.58	1.44	1.56	0.50
Inventiones Mathematicae (1966 -)	0.22	0.22	--	1.15	1.14	1.25	1.69	1.78	1.00
Journal of the American Math. Society (1988 -)	0.00	0.00	--	0.22	0.22	0.25	0.99	1.06	0.44
Journal of the American Stat. Assn. (1922 -)	0.44	0.44	--	0.64	0.66	0.42	0.42	0.47	0.00
Journal of Computational Physics (1966 -)	0.06	0.06	--	0.22	0.21	0.25	1.32	1.21	2.19
PNAS (1915 -)	1.55	1.55	--	0.23	0.25	0.00	0.37	0.38	0.25
Transactions of the American Math. Soc. (1900 -)	1.94	1.94	--	1.55	1.65	0.58	1.13	1.16	0.94
Cumulative citations in math journals									
Advances in Mathematics (1961 -)	0.03	0.03	--	14.15	15.11	4.75	31.43	33.70	13.25
American Journal of Mathematics (1878 -)	16.77	16.77	--	14.26	15.31	3.92	34.23	37.12	11.13
Annals of Mathematics (1874 -)	41.26	41.26	--	46.72	49.86	15.83	84.42	88.23	54.00
Annals of Probability (1974 -)	0.00	0.00	--	7.33	8.08	0.00	24.32	26.24	8.94
Annals of Statistics (1973 -)	51.99	51.99	--	55.15	57.08	36.17	34.92	39.28	0.00
Acta Mathematica (1882 -)	4.39	4.39	--	12.08	12.87	4.25	21.46	19.33	38.50
Comm. on Pure and Applied Math (1948 -)	8.83	8.83	--	36.75	35.53	48.75	116.86	123.59	63.06
Comm. in Mathematical Physics (1965 -)	0.02	0.02	--	36.67	39.05	13.25	102.56	111.18	33.63
Duke Mathematical Journal (1935 -)	5.93	5.93	--	10.89	11.27	7.17	36.11	39.03	12.75
Inventiones Mathematicae (1966 -)	1.33	1.33	--	26.32	26.92	20.42	67.97	73.03	27.44
Journal of the American Math. Society (1988 -)	0.00	0.00	--	0.03	0.02	0.17	23.83	24.11	21.63
Journal of the American Stat. Assn. (1922 -)	10.65	10.65	--	23.91	25.51	8.17	31.44	35.37	0.00
Journal of Computational Physics (1966 -)	0.09	0.09	--	4.77	4.84	4.08	153.38	130.66	335.06
PNAS (1915 -)	7.84	7.84	--	3.55	3.92	0.00	6.60	7.05	2.94
Transactions of the American Math. Soc. (1900 -)	12.68	12.68	--	15.05	16.39	1.92	27.58	29.91	8.88
Number of years since first publication	17.75	17.75	--	23.10	23.43	19.83	26.46	26.93	22.69
Number of Authors	103	103	--	130	118	12	144	128	16
Unmatched Members	13	13	0	7	6	1	3	2	1

Notes: Table presents characteristics of mathematicians in the years of election as Members of the American Academy of Arts and Sciences.

APPENDIX TABLE S5A PSYCHOLOGY
SUMMARY STATISTICS FOR NAS MEMBERS IN ELECTION YEAR

	Newly Elected Members in Year of Election								
	1960-1979			1980-1999			2000-2019		
	All	Male	Female	All	Male	Female	All	Male	Female
	(1)	(2)	(3)	(4)	(5)	(6)	(7)	(8)	(9)
Percent Female	5	0	100	15.63	0	100	49.09	0	100
Percent Unknown Gender	0	0	0	0	0	0	0	0	0
Percent Members (as of current year)									
Current Member of AAAS (1980+)	15	15.79	0	37.5	37.04	40	49.09	39.29	59.26
Current Member of NAS (1980+)	100	100	100	100	100	100	100	100	100
Cumulative publications in psychology journals									
Psychological Science (1990 -)	0	0	0	0.06	0.07	0	2.65	2.68	2.63
Psychological Review (1984 -)	2.08	2.11	1.5	1.69	1.89	0.6	1.67	2.61	0.7
Psychological Bulletin (1904 -)	0.72	0.71	1	0.56	0.63	0.2	0.35	0.39	0.3
Trends in Cognitive Sciences (1997 -)	0	0	0	0	0	0	1.13	1.21	1.04
J. of Experimental Psych: General (1975 -)	5.88	5.92	5	3.66	4.04	1.6	1.6	1.93	1.26
J. of Personality and Social Psychology (1965 -)	0.3	0.32	0	0.44	0.41	0.6	4.36	4.57	4.15
Annual Review of Psychology (1950 -)	0.42	0.39	1	0.38	0.41	0.2	0.58	0.64	0.52
American Psychologist (1946 -)	0.9	0.89	1	0.63	0.63	0.6	1.11	0.93	1.3
Child Development (1930 -)	0.07	0.08	0	1.91	1.37	4.8	2.8	1.64	4
Cognition (1972 -)	0.03	0.03	0	0.88	0.59	2.4	3.73	3.54	3.93
Cognitive Psychology (1970 -)	0.23	0.21	0.5	0.84	0.74	1.4	1.51	1.32	1.7
Developmental Psychology (1969 -)	0	0	0	0.69	0.44	2	1.69	1.64	1.74
PNAS (1915 -)	0	0	0	0	0	0	0.35	0.5	0.19
Cumulative citations in psychology journals									
Psychological Science (1990 -)	0	0	0	0.19	0.22	0	207.73	199.43	216.33
Psychological Review (1984 -)	137.05	133.92	196.5	384.44	428.81	144.8	792.56	893.75	687.63
Psychological Bulletin (1904 -)	48.05	48.58	38	112.09	116.93	86	145.44	51.89	242.44
Trends in Cognitive Sciences (1997 -)	0	0	0	0	0	0	154.18	212.21	94
J. of Experimental Psych: General (1975 -)	138.68	139.03	132	201.81	222.3	91.2	156.76	216.93	94.37
J. of Personality and Social Psychology (1965 -)	5.53	5.82	0	13.41	11.93	21.4	783.67	596.18	978.11
Annual Review of Psychology (1950 -)	4.42	3.95	13.5	20.06	22.93	4.6	124.04	136	111.63
American Psychologist (1946 -)	25.98	27.13	4	38.47	41.93	19.8	252.25	183.96	323.07
Child Development (1930 -)	0	0	0	20	10.78	69.8	114.98	106.93	123.33
Cognition (1972 -)	0.03	0.03	0	26.84	11.67	108.8	331.16	292.29	371.48
Cognitive Psychology (1970 -)	4.7	4.95	0	90.78	61.04	251.4	186.55	249.54	121.22
Developmental Psychology (1969 -)	0	0	0	17.69	12.89	43.6	104.62	118.61	90.11
PNAS (2009 -)	0	0	0	0	0	0	8.09	13.64	2.33
Number of years since first publication	18.95	18.42	29	23.53	23.3	24.8	30.2	33.07	27.22
Number of Authors	40	38	2	32	27	5	55	28	27
Unmatched Members	1	1	0	6	4	2	2	2	0

Notes: Table presents characteristics of psychologists in the years of election as Members of the National Academy of Sciences.

APPENDIX TABLE S5B ECONOMICS
SUMMARY STATISTICS FOR NAS MEMBERS IN ELECTION YEAR

	Newly Elected Members in Year of Election								
	1960-1979			1980-1999			2000-2019		
	All	Male	Female	All	Male	Female	All	Male	Female
	(1)	(2)	(3)	(4)	(5)	(6)	(7)	(8)	(9)
Percent Female	0	0	--	5.26	0	100	14.58	0	100
Percent Unknown Gender	0	0	--	0	0	0	0	0	0
Percent Members (as of current year)									
Current Member of AAAS (1980+)	55.56	55.56	--	92.11	94.44	50	95.83	95.12	100
Current Member of NAS (1980+)	100	100	--	100	100	100	100	100	100
Cumulative publications in Economics journals									
American Economic Review	2.63	2.63	--	2.53	2.61	1	4.17	4.27	3.57
Econometrica	4.41	4.41	--	3.05	3.22	0	4.13	4.54	1.71
Journal of Political Economy	3.22	3.22	--	2.16	2.19	1.5	3.1	3.37	1.57
Quarterly Journal of Economics	1.56	1.56	--	1.58	1.61	1	3.56	3.44	4.29
Review of Economic Studies	1.7	1.7	--	2.05	2.17	0	3.15	3.51	1
Journal of Econometrics	0	0	--	0.32	0.33	0	1.04	1.22	0
Journal of Economic Theory	0.37	0.37	--	2.11	2.22	0	2.81	3.22	0.43
RAND Journal of Economics	0.26	0.26	--	0.95	1	0	1.19	1.24	0.86
Journal of Economic Perspectives	0	0	--	0.21	0.19	0.5	1.81	1.73	2.29
Journal of Economic Literature	0.04	0.04	--	0.32	0.31	0.5	0.6	0.56	0.86
Review of Economics and Statistics	2.07	2.07	--	0.71	0.75	0	0.69	0.61	1.14
Journal of Finance	0.07	0.07	--	0.32	0.33	0	0.71	0.83	0
Journal of Monetary Economics	0	0	--	0.24	0.25	0	0.56	0.63	0.14
Journal of Public Economics	0	0	--	0.97	1.03	0	1.58	1.54	1.86
Cumulative citations in econ journals									
American Economic Review (1911 -)	35.96	35.96	--	153.05	150.25	203.5	579.35	613.37	380.14
Econometrica (1933 -)	90.74	90.74	--	195.95	206.83	0	610.35	683.17	183.86
Journal of Political Economy (1892 -)	49.3	49.3	--	134.45	140.08	33	588.35	664.88	140.14
Quarterly Journal of Economics (1886 -)	12.22	12.22	--	52.55	54.53	17	670.44	644.71	821.14
Review of Economic Studies (1933 -)	15.37	15.37	--	56.5	59.64	0	326.85	375.02	44.71
Journal of Econometrics (1973 -)	0	0	--	5.97	6.31	0	212.1	248.32	0
Journal of Economic Theory (1968 -)	1.59	1.59	--	118.03	124.58	0	163	183.2	44.71
RAND Journal of Economics (1970 -)	0	0	--	0.68	0.72	0	70.83	76.66	36.71
Journal of Economic Perspectives (1987 -)	0	0	--	0.76	0.81	0	134.75	125.22	190.57
Journal of Economic Literature (1963 -)	1.44	1.44	--	17.71	18.25	8	100.23	78.56	227.14
Review of Economics and Statistics (1919 -)	40.11	40.11	--	8.5	8.97	0	61.38	64.39	43.71
Journal of Finance (1946 -)	0.11	0.11	--	16.24	17.14	0	137.44	160.9	0
Journal of Monetary Economics (1973 -)	0	0	--	6.13	6.47	0	52.77	54.73	41.29
Journal of Public Economics (1972 -)	0	0	--	11.79	12.44	0	62.88	54.41	112.43
Number of years since first publication	26	26	--	29.87	30.25	23	32.5	34.41	21.29
Number of Authors	27	27	--	38	36	2	48	41	7
Unmatched Members	1	1	0	0	0	0	0	0	0

Notes: Table presents characteristics of economists in the years of election as Members of the National Academy of Sciences.

APPENDIX TABLE S5C MATHEMATICS
SUMMARY STATISTICS FOR NAS MEMBERS IN ELECTION YEAR

	Newly Elected Members in Year of Election								
	1960-1979			1980-1999			2000-2019		
	All	Male	Female	All	Male	Female	All	Male	Female
	(1)	(2)	(3)	(4)	(5)	(6)	(7)	(8)	(9)
Percent Female	2.67	0	100	5.15	0	100	12.17	0	100
Percent Unknown Gender	0	0	0	0	0	0	0	0	0
Percent Members (as of current year)									
Current Member of AAAS (1980+)	37.33	38.36	0	55.67	53.26	100	49.57	50.5	42.86
Current Member of NAS (1980+)	100	100	100	100	100	100	100	100	100
Cumulative publications in mathematics journals									
Advances in Mathematics (1961 -)	0.17	0.18	0	0.81	0.85	0.2	0.76	0.8	0.43
American Journal of Mathematics (1878 -)	2.17	2.23	0	1.11	1.15	0.4	0.51	0.5	0.64
Annals of Mathematics (1874 -)	3.29	3.36	1	1.81	1.84	1.4	1.5	1.61	0.71
Annals of Probability (1974 -)	0	0	0	0.61	0.64	0	1.77	1.9	0.79
Annals of Statistics (1973 -)	3.27	3.34	0.5	2.1	2.22	0	2.54	2.72	1.21
Acta Mathematica (1882 -)	0.51	0.52	0	0.52	0.51	0.6	0.42	0.45	0.21
Comm. on Pure and Applied Math (1948 -)	1.84	1.89	0	1.55	1.4	4.2	1.23	1.28	0.86
Comm. in Mathematical Physics (1965 -)	0.04	0.04	0	1.46	1.47	1.4	2.36	2.5	1.36
Duke Mathematical Journal (1935 -)	1.27	1.3	0	0.62	0.63	0.4	1.18	1.27	0.57
Inventiones Mathematicae (1966 -)	0.41	0.42	0	1.25	1.21	2	1.65	1.8	0.57
Journal of the American Math. Society (1988 -)	0	0	0	0.22	0.22	0.2	0.79	0.88	0.14
Journal of the American Stat. Assn. (1922 -)	0.49	0.48	1	0.47	0.5	0	1.46	1.46	1.5
Journal of Computational Physics (1966 -)	0	0	0	0.34	0.35	0.2	1.53	1.52	1.57
PNAS (1915 -)	1.77	1.82	0	0.48	0.51	0	0.35	0.37	0.21
Transactions of the American Math. Soc. (1900 -)	1.95	1.99	0.5	1.69	1.74	0.8	1.1	1.11	1.07
Cumulative citations in mathematics journals									
Advances in Mathematics (1961 -)	0.4	0.41	0	15.91	16.76	0.2	50.21	53.91	23.5
American Journal of Mathematics (1878 -)	18.4	18.9	0	25.78	26.92	4.8	19.3	19.67	16.57
Annals of Mathematics (1874 -)	46.89	47.52	24	64.44	66.51	26.4	89.16	96.23	38.14
Annals of Probability (1974 -)	0	0	0	8.12	8.57	0	43.09	46.43	19
Annals of Statistics (1973 -)	63.09	64.75	2.5	50.13	52.86	0	230.57	255.74	48.93
Acta Mathematica (1882 -)	11.92	12.25	0	12.29	12.61	6.4	17.97	19.41	7.57
Comm. on Pure and Applied Math (1948 -)	22.55	23.16	0	71.87	57.87	329.4	96.78	103.47	48.57
Comm. in Mathematical Physics (1965 -)	0.37	0.38	0	32.63	33.02	25.4	113.88	120.28	67.71
Duke Mathematical Journal (1935 -)	8.84	9.08	0	5.66	5.67	5.4	39.28	42.83	13.64
Inventiones Mathematicae (1966 -)	2.73	2.81	0	29.67	30.04	22.8	90.14	99.04	25.93
Journal of the American Math. Society (1988 -)	0	0	0	0.03	0.03	0	28.03	31.27	4.64
Journal of the American Stat. Assn. (1922 -)	6.07	6.21	1	15.3	16.13	0	163.29	171.5	104
Journal of Computational Physics (1966 -)	0	0	0	7.13	7.4	2.2	179.59	184.87	141.5
PNAS (1915 -)	11.31	11.62	0	7.62	8.03	0	116.96	133.03	1
Transactions of the American Math. Soc. (1900 -)	12.56	12.74	6	22.27	23.25	4.2	29.2	31.25	14.43
Number of years since first publication	21.8	21.58	30	24.52	24.66	21.8	27.89	28.19	25.71
Number of Authors	75	73	2	97	92	5	115	101	14
Unmatched Members	6	6	0	5	5	0	5	3	2

Notes: Table presents characteristics of mathematicians in the years of election as Members of the National Academy of Sciences.

APPENDIX TABLE S6A PSYCHOLOGY
PREDICTORS OF SELECTION AS AAAS/NAS MEMBERS

	Logit Regression for Selection as AAAS Member in Year t:			Logit Regression for Selection as NAS Member in Year t:		
	1960-1979	1980-1999	2000-2019	1960-1979	1980-1999	2000-2019
	(1)	(2)	(3)	(4)	(5)	(6)
Female × (1960-79)	-0.738			-0.701		
	(0.512)			(0.756)		
Female × (1980-89)		-0.069			-0.496	
		(0.630)			(0.758)	
Female × (1990-99)		0.702			0.480	
		(0.362)			(0.774)	
Female × (2000-09)			1.171			1.049
			(0.317)			(0.428)
Female × (2010-19)			1.301			1.279
			(0.348)			(0.422)
Cumulative Publications and Citations Received in Psychology journals						
Cumulative cites in all journals	-0.376	0.246	0.025	-0.246	0.218	0.463
	(0.165)	(0.217)	(0.268)	(0.225)	(0.252)	(0.415)
Psychological Science	0.000	0.688	0.082	0.000	-0.021	0.059
	(.)	(0.440)	(0.057)	(.)	(0.827)	(0.087)
Cumulative citations in PS	0.000	-0.215	0.267	0.000	-0.126	0.141
	(.)	(0.246)	(0.066)	(.)	(0.205)	(0.096)
Psychological Review	-0.181	0.301	-0.057	-0.017	0.238	0.080
	(0.138)	(0.084)	(0.129)	(0.113)	(0.166)	(0.078)
Cumulative citations in PR	0.417	0.082	0.055	0.524	0.066	0.001
	(0.121)	(0.062)	(0.051)	(0.192)	(0.111)	(0.067)
Psychological Bulletin	0.027	-0.275	0.058	-0.185	-0.720	-0.084
	(0.231)	(0.196)	(0.050)	(0.260)	(0.401)	(0.173)
Cumulative citations in PB	-0.227	-0.007	-0.016	0.069	0.221	-0.154
	(0.120)	(0.080)	(0.047)	(0.140)	(0.147)	(0.083)
Trends in Cognitive Sciences	0.000	0.000	-0.103	0.000	0.000	-0.032
	(.)	(.)	(0.176)	(.)	(.)	(0.128)
Cumulative citations in TiCS	0.000	0.000	0.163	0.000	0.000	0.283
	(.)	(.)	(0.078)	(.)	(.)	(0.086)
Journal of Experimental Psychology: General	-0.125	-0.118	0.003	0.036	-0.116	0.065
	(0.064)	(0.046)	(0.070)	(0.030)	(0.045)	(0.069)
Cumulative citations in JEPG	0.020	0.149	0.021	0.032	0.208	-0.056
	(0.096)	(0.071)	(0.066)	(0.120)	(0.117)	(0.085)
Journal of Personality and Social Psychology	0.029	0.021	0.082	0.131	-0.255	0.042
	(0.076)	(0.025)	(0.014)	(0.093)	(0.092)	(0.030)
Cumulative citations in JPSP	0.282	0.059	0.015	-0.305	0.027	-0.071
	(0.131)	(0.072)	(0.056)	(0.182)	(0.141)	(0.078)
Annual Review of Psychology	1.024	-0.774	0.531	-0.653	-1.572	0.578
	(0.554)	(0.531)	(0.382)	(0.553)	(0.641)	(0.441)
Cumulative citations in ARP	-0.299	0.283	0.015	0.566	0.595	-0.085
	(0.326)	(0.143)	(0.089)	(0.220)	(0.177)	(0.112)
American Psychologist	0.087	-0.002	0.098	-0.223	-0.099	-0.022
	(0.133)	(0.121)	(0.077)	(0.239)	(0.179)	(0.084)
Cumulative citations in AP	0.457	0.249	0.122	0.527	0.027	0.210
	(0.123)	(0.080)	(0.057)	(0.172)	(0.142)	(0.087)
Child Development	0.140	0.133	0.042	-0.286	0.138	0.016
	(0.092)	(0.068)	(0.035)	(0.449)	(0.062)	(0.042)

APPENDIX TABLE S6A PSYCHOLOGY (CONTINUED)

Cumulative citations in CD	0.049	-0.100	0.016	0.000	-0.279	-0.036
	(0.183)	(0.116)	(0.066)	(.)	(0.199)	(0.088)
Cognition	1.650	0.232	0.156	0.293	0.615	0.161
	(1.125)	(0.103)	(0.060)	(0.732)	(0.177)	(0.055)
Cumulative citations in Cog	0.000	0.152	0.105	0.300	-0.337	0.034
	(.)	(0.119)	(0.079)	(0.244)	(0.204)	(0.102)
Cognitive Psychology	0.309	0.098	0.009	1.632	0.319	-0.050
	(0.869)	(0.128)	(0.137)	(0.457)	(0.252)	(0.140)
Cumulative citations in CP	0.462	0.115	0.102	-0.269	-0.067	0.151
	(0.341)	(0.088)	(0.085)	(0.367)	(0.196)	(0.095)
Development Psychology	0.000	-0.096	0.031	0.000	0.038	0.002
	(.)	(0.152)	(0.038)	(.)	(0.202)	(0.055)
Cumulative citations in DP	0.000	0.079	0.040	0.000	-0.142	-0.097
	(.)	(0.110)	(0.060)	(.)	(0.198)	(0.078)
Proceedings of the National Academy of Sciences	0.000	0.000	-0.012	0.000	0.000	-0.172
	(.)	(.)	(0.319)	(.)	(.)	(0.385)
Cumulative citations in PNAS	0.000	0.000	-0.058	0.000	0.000	0.101
	(.)	(.)	(0.133)	(.)	(.)	(0.174)
Years since first publication (Omitted: < 10 years)						
10-19 years	0.606	-0.077	0.106	0.986	-1.034	0.305
	(0.389)	(0.433)	(0.830)	(0.431)	(0.588)	(0.640)
20-29 years	1.117	0.974	1.609	0.924	-1.517	1.143
	(0.520)	(0.549)	(0.814)	(0.563)	(0.928)	(0.657)
30+ years	1.749	1.418	1.818	0.864	0.902	1.319
	(0.631)	(0.632)	(0.849)	(0.835)	(0.748)	(0.663)
Levels of Top 5 Citations (Omitted: < 90th Percentile)						
90th-95th percentile	1.917	0.377	0.753	0.515	0.441	0.346
	(0.669)	(0.581)	(0.561)	(0.925)	(0.915)	(0.779)
95th-99th percentile	2.261	0.695	1.030	1.068	1.023	0.286
	(0.787)	(0.666)	(0.658)	(1.043)	(0.844)	(1.005)
99th-99.5th percentile	2.666	1.278	1.405	0.808	2.245	0.210
	(1.069)	(0.828)	(0.830)	(1.426)	(1.112)	(1.308)
99.5th+ percentile	3.821	1.241	1.486	1.176	2.812	1.212
	(0.977)	(0.881)	(0.947)	(1.521)	(1.146)	(1.400)
N	112,011	274,946	395,070	102,935	233,021	396,130
Pseudo R-squared	0.261	0.309	0.375	0.291	0.324	0.354

Notes: Standard errors, clustered by author, in parentheses. Table entries are logistic regression coefficients: models are fit to set of active economists in a given year who are not yet members of the AAAS or NAS. Researchers with unknown gender are excluded from the sample. Measures of publications and asinh of citations represent numbers of papers published, and asinh of citations received, up to current year. All models include year fixed effects.

APPENDIX TABLE S6B ECONOMICS
PREDICTORS OF SELECTION AS AAAS/NAS MEMBERS

	Logit Regression for Selection as AAAS Member in Year t:			Logit Regression for Selection as NAS Member in Year t:		
	1960-1979	1980-1999	2000-2019	1960-1979	1980-1999	2000-2019
	(1)	(2)	(3)	(4)	(5)	(6)
Female × (1960-79)	-0.073			0.000		
	(0.635)			(.)		
Female × (1980-89)		1.803			2.097	
		(0.634)			(1.094)	
Female × (1990-99)		0.625			1.805	
		(0.659)			(1.019)	
Female × (2000-09)			1.304			2.848
			(0.446)			(0.786)
Female × (2010-19)			1.989			2.745
			(0.323)			(0.579)
Cumulative Publications and Citations Received in Eco	0.439	0.861	0.915			
Cumulative cites in all journals	(0.173)	(0.313)	(0.247)	1.215	1.357	1.219
				(0.804)	(0.610)	(0.471)
American Economic Review	0.132	0.060	0.106	-0.080	0.016	0.121
	(0.101)	(0.092)	(0.072)	(0.248)	(0.135)	(0.076)
Cumulative citations in AER	0.042	-0.045	0.001	0.051	-0.036	-0.077
	(0.106)	(0.065)	(0.052)	(0.217)	(0.120)	(0.092)
Econometrica	0.135	0.101	0.205	0.046	-0.039	0.060
	(0.081)	(0.045)	(0.042)	(0.080)	(0.078)	(0.052)
Cumulative citations in ECTA	0.013	0.054	0.144	0.348	0.101	0.132
	(0.101)	(0.057)	(0.044)	(0.203)	(0.110)	(0.081)
Journal of Political Economy	0.005	0.080	0.169	0.231	0.071	0.053
	(0.079)	(0.068)	(0.055)	(0.136)	(0.122)	(0.089)
Cumulative citations in JPE	0.042	0.045	0.044	-0.189	-0.157	0.054
	(0.102)	(0.063)	(0.048)	(0.186)	(0.129)	(0.090)
Quarterly Journal of Economics	-0.132	0.089	0.395	0.158	-0.070	-0.006
	(0.120)	(0.115)	(0.069)	(0.239)	(0.248)	(0.098)
Cumulative citations in QJE	0.095	0.105	0.042	-0.380	-0.104	0.094
	(0.132)	(0.080)	(0.054)	(0.212)	(0.185)	(0.094)
Review of Economic Studies	0.147	-0.057	0.058	-0.043	-0.037	-0.026
	(0.109)	(0.100)	(0.084)	(0.111)	(0.126)	(0.118)
Cumulative citations in REStud	0.113	0.166	0.040	0.214	0.088	0.209
	(0.134)	(0.074)	(0.062)	(0.154)	(0.116)	(0.105)
Journal of Econometrics	0.209	-0.007	-0.088	0.000	-0.320	-0.051
	(0.197)	(0.072)	(0.076)	(.)	(0.295)	(0.062)
Cumulative citations in JE	0.000	0.032	0.046	0.000	0.215	-0.073
	(.)	(0.104)	(0.079)	(.)	(0.328)	(0.110)
Journal of Economic Theory	0.045	-0.018	-0.011	-0.617	0.089	0.088
	(0.249)	(0.056)	(0.036)	(0.753)	(0.086)	(0.060)
Cumulative citations in JET	-0.208	0.099	0.125	0.743	0.150	-0.041
	(0.252)	(0.060)	(0.059)	(0.459)	(0.118)	(0.096)
RAND Journal of Economics	0.317	0.155	-0.201	-0.383	0.168	-0.138
	(0.243)	(0.116)	(0.100)	(0.566)	(0.146)	(0.069)
Cumulative citations in RAND	0.000	-0.089	0.043	0.000	-0.178	-0.035
	(.)	(0.109)	(0.067)	(.)	(0.228)	(0.087)
Journal of Economic Perspectives	0.000	0.167	0.286	0.000	0.448	0.118
	(.)	(0.097)	(0.079)	(.)	(0.221)	(0.051)

APPENDIX TABLE S6B ECONOMICS (CONTINUED)

Cumulative citations in JEP	0.000	0.166	0.093	0.000	-0.263	0.097
	(.)	(0.130)	(0.050)	(.)	(0.354)	(0.088)
Journal of Economic Literature	1.496	0.220	0.173	-6.363	0.607	0.744
	(0.715)	(0.452)	(0.274)	(3.478)	(0.713)	(0.424)
Cumulative citations in JEL	-0.341	0.008	-0.036	1.539	0.024	-0.106
	(0.354)	(0.146)	(0.073)	(0.897)	(0.197)	(0.132)
Review of Economics and Statistics	-0.017	-0.494	-0.204	0.024	-0.005	-0.418
	(0.113)	(0.217)	(0.112)	(0.181)	(0.326)	(0.225)
Cumulative citations in REStat	0.105	0.160	-0.003	0.176	-0.161	0.198
	(0.117)	(0.104)	(0.068)	(0.199)	(0.249)	(0.126)
Journal of Finance	0.015	0.005	0.164	0.079	-1.149	-0.240
	(0.228)	(0.113)	(0.040)	(0.511)	(0.723)	(0.087)
Cumulative citations in JF	-0.419	-0.152	-0.205	-0.400	0.570	0.160
	(0.355)	(0.105)	(0.056)	(0.605)	(0.245)	(0.105)
Journal of Monetary Economics	0.000	-0.254	-0.079	0.000	-0.303	-0.283
	(.)	(0.132)	(0.090)	(.)	(0.311)	(0.162)
Cumulative citations in JME	0.000	0.231	0.025	0.000	0.225	0.045
	(.)	(0.101)	(0.069)	(.)	(0.224)	(0.125)
Journal of Public Economics	0.347	0.181	0.009	0.000	0.207	0.024
	(0.174)	(0.133)	(0.091)	(.)	(0.187)	(0.088)
Cumulative citations in JPubE	-0.520	-0.226	-0.121	0.000	-0.228	-0.014
	(0.361)	(0.133)	(0.071)	(.)	(0.246)	(0.105)
Years since first publication (Omitted: < 10 years)	1.232	0.922	0.086			
10-19 years	(0.386)	(0.534)	(0.513)	-0.554	12.871	11.531
	2.056	1.127	-0.464	(0.826)	(0.746)	(0.735)
20-29 years	(0.413)	(0.571)	(0.539)	-0.117	13.788	11.804
	2.082	1.754	-1.037	(0.793)	(0.811)	(0.834)
30+ years	(0.502)	(0.627)	(0.598)	1.340	15.493	12.549
	0.000	0.000	0.000	(0.984)	(0.870)	(0.864)
Levels of Top 5 Citations (Omitted: < 90th Percentile)	(.)	(.)	(.)			
90th-95th percentile	0.455	-0.200	0.800	-0.948	-2.384	0.252
	(0.400)	(0.506)	(0.507)	(1.403)	(1.112)	(0.913)
95th-99th percentile	0.671	0.472	0.661	-0.118	-1.132	0.004
	(0.455)	(0.594)	(0.654)	(1.645)	(1.063)	(1.058)
99th-99.5th percentile	0.254	0.787	0.932	0.944	-1.168	0.213
	(0.697)	(0.775)	(0.798)	(1.900)	(1.471)	(1.283)
99.5th+ percentile	0.758	0.950	0.276	0.528	-0.214	0.002
	(0.688)	(0.937)	(0.895)	(2.306)	(1.613)	(1.492)
N	52566	136993	220753	33,046	138,853	223,889
Pseudo R-squared	0.263	0.370	0.431	0.503	0.391	0.433

Notes: Standard errors, clustered by author, in parentheses. Table entries are logistic regression coefficients: models are fit to set of active economists in a given year who are not yet members of the AAAS or NAS. Researchers with unknown gender are excluded from the sample. Measures of publications and asinh of citations represent numbers of papers published, and asinh of citations received, up to current year. All models include year fixed effects.

APPENDIX TABLE S6C MATHEMATICS
PREDICTORS OF SELECTION AS AAAS/NAS MEMBERS

	Logit Regression for Selection as AAAS Member in Year t:			Logit Regression for Selection as NAS Member in Year t:		
	1960-1979	1980-1999	2000-2019	1960-1979	1980-1999	2000-2019
	(1)	(2)	(3)	(4)	(5)	(6)
Female × (1960-79)	0.000			0.771		
	(.)			(0.752)		
Female × (1980-89)		1.246			0.201	
		(0.592)			(1.112)	
Female × (1990-99)		1.895			1.546	
		(0.380)			(0.556)	
Female × (2000-09)			0.841			1.538
			(0.479)			(0.502)
Female × (2010-19)			1.968			1.744
			(0.352)			(0.384)
Cumulative Publications and Citations Received in Math journals						
Cumulative cites in all journals	0.063	0.517	0.397	0.023	0.347	1.108
	(0.172)	(0.229)	(0.212)	(0.218)	(0.272)	(0.315)
Advances in Mathematics	1.051	0.021	0.151	0.289	0.153	0.025
	(0.329)	(0.123)	(0.062)	(0.386)	(0.128)	(0.088)
Citations in AM	-1.409	0.166	-0.100	0.225	0.059	0.012
	(0.731)	(0.100)	(0.073)	(0.420)	(0.119)	(0.097)
American Journal of Mathematics	-0.017	-0.121	-0.086	-0.009	-0.018	-0.390
	(0.025)	(0.092)	(0.095)	(0.019)	(0.070)	(0.208)
Citations in AJM	0.214	0.096	0.153	0.136	0.052	0.057
	(0.114)	(0.095)	(0.077)	(0.104)	(0.097)	(0.116)
Annals of Mathematics	-0.021	-0.004	0.112	0.032	0.081	0.042
	(0.031)	(0.057)	(0.061)	(0.040)	(0.071)	(0.060)
Citations in AM	0.488	0.197	0.111	0.211	0.052	0.205
	(0.130)	(0.071)	(0.055)	(0.101)	(0.087)	(0.072)
Annals of Probability	0.874	-0.114	0.016	0.000	-0.145	0.031
	(0.323)	(0.096)	(0.035)	(.)	(0.087)	(0.034)
Citations in AP	0.000	0.250	0.131	0.000	0.408	0.129
	(.)	(0.120)	(0.081)	(.)	(0.118)	(0.083)
Annals of Statistics	0.080	0.057	0.021	0.080	0.058	0.007
	(0.033)	(0.025)	(0.030)	(0.036)	(0.040)	(0.012)
Citations in AP	0.044	0.049	-0.001	0.048	-0.010	0.103
	(0.122)	(0.078)	(0.070)	(0.129)	(0.101)	(0.060)
Acta Mathematica	0.410	0.343	-0.273	-0.174	-0.253	0.137
	(0.127)	(0.123)	(0.245)	(0.203)	(0.208)	(0.181)
Citations in ActaM	-0.199	-0.097	0.078	0.296	0.199	-0.114
	(0.148)	(0.097)	(0.092)	(0.159)	(0.106)	(0.115)
Communications on Pure and Applied Math	0.150	0.075	0.088	0.038	0.062	0.044
	(0.058)	(0.034)	(0.032)	(0.060)	(0.047)	(0.034)
Citations in CM	0.141	0.127	0.128	0.276	0.125	0.078
	(0.146)	(0.070)	(0.048)	(0.114)	(0.084)	(0.068)
Communications in Mathematical Physics	0.054	-0.019	-0.032	-0.756	0.005	0.001
	(0.181)	(0.038)	(0.024)	(0.578)	(0.037)	(0.028)
Citations in CMP	-0.449	-0.015	0.087	0.645	0.046	-0.058
	(0.196)	(0.077)	(0.048)	(0.527)	(0.087)	(0.063)
Duke Mathematical Journal	-0.029	-0.221	-0.087	-0.006	-0.485	0.033
	(0.052)	(0.124)	(0.106)	(0.030)	(0.168)	(0.099)

APPENDIX TABLE S6C MATHEMATICS (CONTINUED)

Citations in DMP	0.133	0.184	0.091	0.107	0.236	-0.007
	(0.111)	(0.089)	(0.074)	(0.105)	(0.114)	(0.094)
Inventiones Mathematicae	0.339	0.127	-0.022	0.379	0.016	0.029
	(0.277)	(0.107)	(0.090)	(0.368)	(0.114)	(0.100)
Citations in IM	0.013	0.044	0.063	0.290	0.205	0.045
	(0.209)	(0.099)	(0.071)	(0.384)	(0.112)	(0.090)
Journal of the American Mathematical Society	0.000	0.616	0.391	0.000	0.839	0.516
	(.)	(0.205)	(0.116)	(.)	(0.196)	(0.151)
Citations in AMS	0.000	-0.593	0.191	0.000	-0.811	0.013
	(.)	(0.383)	(0.068)	(.)	(0.555)	(0.085)
Journal of the American Statistical Association	-0.097	-0.099	0.044	0.064	-0.145	0.018
	(0.073)	(0.094)	(0.063)	(0.088)	(0.173)	(0.026)
Citations in AMA	0.280	0.064	-0.153	-0.013	0.110	-0.096
	(0.122)	(0.105)	(0.096)	(0.173)	(0.150)	(0.075)
Journal of Computational Physics	0.116	0.054	0.038	0.000	0.063	0.017
	(0.301)	(0.110)	(0.013)	(.)	(0.070)	(0.012)
Citations in JCP	0.496	-0.031	-0.012	0.000	0.098	-0.007
	(0.606)	(0.125)	(0.065)	(.)	(0.111)	(0.067)
Proceedings of the National Academy of Sciences	0.019	-0.066	0.045	-0.013	0.081	0.316
	(0.038)	(0.170)	(0.062)	(0.035)	(0.149)	(0.156)
Citations in PNAS	-0.098	0.088	-0.022	0.175	0.192	-0.055
	(0.126)	(0.138)	(0.106)	(0.135)	(0.169)	(0.117)
Transactions of American Math. Soc.	0.023	0.108	-0.092	-0.067	0.064	-0.049
	(0.082)	(0.039)	(0.105)	(0.072)	(0.093)	(0.065)
Citations in TAMS	0.004	-0.158	0.003	0.111	-0.067	-0.057
	(0.125)	(0.073)	(0.083)	(0.105)	(0.101)	(0.074)
Years since first publication (Omitted: < 10 years)						
10-19 years	-0.130	-0.375	0.677	0.580	-0.108	-0.406
	(0.314)	(0.332)	(0.390)	(0.456)	(0.481)	(0.448)
20-29 years	-0.565	-0.042	0.714	1.027	0.697	-0.325
	(0.404)	(0.406)	(0.402)	(0.497)	(0.538)	(0.469)
30+ years	-0.492	-0.134	0.933	0.707	0.812	-0.020
	(0.419)	(0.483)	(0.439)	(0.579)	(0.661)	(0.499)
Levels of Top 5 Citations (Omitted: < 90th Percentile)						
90th-95th percentile	0.415	1.085	1.033	1.176	0.682	-0.222
	(0.515)	(0.483)	(0.441)	(0.629)	(0.579)	(0.500)
95th-99th percentile	0.923	1.207	1.431	1.570	1.253	0.094
	(0.579)	(0.605)	(0.540)	(0.694)	(0.683)	(0.668)
99th-99.5th percentile	0.799	1.336	0.926	1.764	1.849	0.266
	(0.755)	(0.774)	(0.748)	(0.931)	(0.837)	(0.858)
99.5th+ percentile	2.139	1.346	1.179	1.885	1.252	-0.090
	(0.812)	(0.864)	(0.835)	(1.056)	(0.993)	(1.098)
N	87,964	217,891	392,099	88,048	218,619	392,935
Pseudo R-squared	0.236	0.263	0.297	0.292	0.282	0.288

Notes: Standard errors, clustered by author, in parentheses. Table entries are logistic regression coefficients: models are fit to set of active economists in a given year who are not yet members of the AAAS or NAS. Researchers with unknown gender are excluded from the sample. Measures of publications and asinh of citations represent numbers of papers published, and asinh of citations received, up to current year. All models include year fixed effects.

APPENDIX TABLE S7: GENDER COEFFICIENT ROBUSTNESS

	Psychology		Econ		Math	
Percent Female (active publishers in 2010-2019)	48.22%		16.72%		10.85%	
	AAAS	NAS	AAAS	NAS	AAAS	NAS
Panel A (no citations)						
Female Coefficient	-0.835	-0.768	-0.352	--	--	0.073
1960-1979	(0.529)	(0.778)	(0.688)	--	--	(0.726)
Female Coefficient	-0.228	-0.767	1.001*	1.210	0.747	-0.209
1980-1989	(0.645)	(0.779)	(0.595)	(1.077)	(0.584)	(1.033)
Female Coefficient	0.811**	0.050	0.201	1.090	1.468***	1.166**
1990-1999	(0.387)	(0.837)	(0.719)	(1.011)	(0.374)	(0.519)
Female Coefficient	1.041***	0.910*	1.033**	1.743**	0.393	1.005**
2000-2009	(0.322)	(0.474)	(0.441)	(0.791)	(0.513)	(0.482)
Female Coefficient	1.394***	1.298***	1.749***	2.217***	1.436***	1.348***
2010-2019	(0.376)	(0.494)	(0.283)	(0.545)	(0.334)	(0.380)
N	782,287	740,062	410,312	395,788	699,035	699,602
Pseudo R-squared	0.275	0.260	0.307	0.331	0.202	0.222
Panel B						
Female Coefficient	-0.581	-0.708	-0.284	--	--	0.578
1960-1979	(0.491)	(0.724)	(0.656)	--	--	(0.740)
Female Coefficient	-0.289	-0.445	1.057*	1.007	0.890	-0.060
1980-1989	(0.629)	(0.775)	(0.601)	(1.084)	(0.598)	(1.029)
Female Coefficient	0.560	0.390	0.174	0.902	1.665***	1.167**
1990-1999	(0.357)	(0.702)	(0.660)	(1.106)	(0.366)	(0.546)
Female Coefficient	0.965***	0.801*	1.129***	1.936**	0.388	0.963**
2000-2009	(0.317)	(0.433)	(0.436)	(0.816)	(0.489)	(0.487)
Female Coefficient	1.319***	1.136***	2.196***	2.406***	1.727***	1.417***
2010-2019	(0.364)	(0.437)	(0.318)	(0.589)	(0.361)	(0.385)
N	782,027	732,086	410,312	395,788	697,954	699,602
Pseudo R-squared	0.309	0.309	0.338	0.382	0.254	0.260

*** p<0.01, ** p<0.05, * p<0.1

Notes: Standard errors, clustered by author, in parentheses. Table entries are logistic regression coefficients: models are fit to to the set of active psychologists, economists, and mathematicians in a given year who are not yet members of the American Academy of Arts and Sciences or National Academy of Sciences. In Panel A, we exclude all variables related to citations and include publications of individual journals and decades since first publication. In panel B, we include publications of individual journals, asinh of citations in individual journals, and year fixed effects.

APPENDIX TABLE S8: POOLED GENDER COEFFICIENTS ACROSS FIELDS AND TIME

	Psychology	Econ	Math
Logit Regression for Selectionas Member in Year t			
Female Coefficient 1960-1979	-0.726	-0.180	-0.406
	(0.455)	(0.617)	(0.719)
Female Coefficient 1980-1989	-0.242	1.870***	0.875
	(0.502)	(0.554)	(0.616)
Female Coefficient 1990-1999	0.663*	0.909	1.770***
	(0.347)	(0.580)	(0.377)
Female Coefficient 2000-2009	1.134***	1.561***	1.172***
	(0.271)	(0.402)	(0.359)
Female Coefficient 2010-2019	1.294***	2.121***	1.864***
	(0.283)	(0.307)	(0.290)
Observations	1,514,113	807,607	1,402,218
Log-Likelihood	-2231.9	-2604.5	-4105.4
Pseudo R-squared	0.335	0.403	0.286

*** p<0.01, ** p<0.05, * p<0.1

Notes: Standard errors, clustered by author, in parentheses. We stack the samples of AAAS and NAS from Table 2 and perform logistic regressions: models are fit to to the set of active psychologists, economists, and mathematicians in a given year who are not yet members of the American Academy of Arts and Sciences or National Academy of Sciences. We interact each variable from Panel B with the corresponding field of the sample (AAAS or NAS) except for Female Coefficient by decade.
